# Kernel weighted least square approach for imputing missing values of metabolomics data

**DOI:** 10.1038/s41598-021-90654-0

**Published:** 2021-05-27

**Authors:** Nishith Kumar, Md. Aminul Hoque, Masahiro Sugimoto

**Affiliations:** 1 Department of Statistics, Bangabandhu Sheikh Mujibur Rahman Science and Technology University, Gopalganj, Bangladesh; 2 Department of Statistics, University of Rajshahi, Rajshahi, Bangladesh; 3 Health Promotion and Preemptive Medicine, Research and Development Center for Minimally Invasive Therapies, Tokyo Medical University, Shinjuku, Tokyo 160-8402 Japan; 4 Institute for Advanced Biosciences, Keio University, Tsuruoka, 997-0052 Japan

**Keywords:** Computational biology and bioinformatics, Mathematics and computing

## Abstract

Mass spectrometry is a modern and sophisticated high-throughput analytical technique that enables large-scale metabolomic analyses. It yields a high-dimensional large-scale matrix (samples × metabolites) of quantified data that often contain missing cells in the data matrix as well as outliers that originate for several reasons, including technical and biological sources. Although several missing data imputation techniques are described in the literature, all conventional existing techniques only solve the missing value problems. They do not relieve the problems of outliers. Therefore, outliers in the dataset decrease the accuracy of the imputation. We developed a new kernel weight function-based proposed missing data imputation technique that resolves the problems of missing values and outliers. We evaluated the performance of the proposed method and other conventional and recently developed missing imputation techniques using both artificially generated data and experimentally measured data analysis in both the absence and presence of different rates of outliers. Performances based on both artificial data and real metabolomics data indicate the superiority of our proposed kernel weight-based missing data imputation technique to the existing alternatives. For user convenience, an R package of the proposed kernel weight-based missing value imputation technique was developed, which is available at https://github.com/NishithPaul/tWLSA.

## Introduction

Metabolomics datasets produced by mass spectrometry (MS) often contain a wide number of missing cells in the data matrix that can be generated from various sources, including both technological and biological hazards. Generally, there are approximately 10% to 40% missing values in metabolomics datasets^1–3^. The reasons include: (i) the metabolite concentration peak is below the analytical method's detectable threshold; (ii) the metabolite concentration peak is not initially present in the chromatogram; (iii) overlapping signal separation; (iv) deconvolution may give false negatives during the separation of overlapping signals, (v) computational and/or measurement error, (vi) the concentration of the metabolite is present in the sample but vanishes during downstream processing, and (vii) the concentration of a particular metabolite is identified in one sample, but does not exist at a significant concentration in another sample^1,3–6^. These missing values can be categorised as (a) missing completely at random (MCAR), (b) missing at random (MAR), and (c) missing not at random (MNAR). If a missing variable is not related to any observed variable or response it is MCAR. If a missing variable is linked with one or more observed variables, but not to the response, it is MAR. The response associated with missing is MNAR. In metabolomics datasets, if the concentration of a metabolite is not seen in one group of samples, but is present in another group of samples, the missing values most likely occur for a biological reason and can be classified as MNAR. However, if the peak of metabolite concentration is smaller than the analytical method's detection threshold, this missing type is a combination of biological and technological issues and can be considered as MNAR. Finally, MCAR is caused by only technological reasons, for example, errors related to peak picking software, in which the peak was evident but not included in the raw data.

The easiest and most straight forward method of dealing with missing values is the filtering method. In this method, variables^7,8^ or samples^9,10^ are removed. In recent times, this is applicable only when the data matrix includes a greater percentage of missing data. To handle the missing value problem, an alternative approach is the imputation technique. The conventional and widely used missing imputation techniques in different studies and software for imputing missing data are half of the minimum value replacement^2,11^, mean replacement^12^, median replacement^12^, k-nearest neighbour (kNN)^13^, Bayesian principal component analysis (BPCA)^14,15^, probabilistic principal component analysis (PPCA)^16^, zero imputation^17^, multiple imputations with expectation maximisation (EM) algorithm and Monte Carlo Markov chain (MCMC) method^18^, expectation–maximization principal component analysis (EM-PCA)^19^, and random forest (RF) imputation^20^. Recently developed techniques include Gibbs sampler-based left-censored missing value imputation approach (GSimp)^21^, quantile regression imputation of left-censored data (QRILC)^2^, kNN on observations with variable pre-selection (“kNN-obs-sel”)^22^, BayesMetab^23^, robust missing imputation using mean absolute error (rmiMAE)^24^, multivariate imputation by chained equations (MICE)^25^, and others. Several missing imputation techniques are described in the literature. However, the selection of the missing imputation technique has a profound impact on univariate and multivariate (unsupervised and supervised) data analyses and interpretation^1,26–28^. Therefore, the appropriate handling of missing data is very important according to the structure or nature of the original data for downstream analysis. The pattern of metabolomics datasets is very complicated because metabolomics datasets contain outliers^29^, non-normality, and inherent correlation structure^30^. However, the missing value imputation techniques, such as mean, kNN, EM-PCA, PPCA, BPCA, and RF, are sensitive to outliers^25^. All the aforementioned techniques can only handle the problem of missing values. They cannot significantly and simultaneously reduce the outlier problem. This is because the conventional imputation algorithms do not directly consider any outlier-robust function or any outlier identification and substitute algorithms. Furthermore, existing outlier resolving techniques do not consider missing value problems. For these reasons, we have developed a novel kernel-weight-based missing imputation (KMI) method that can simultaneously overcome both the missing value imputation problems and outliers. We compared our proposed method with widely used conventional techniques and recently developed techniques.

To evaluate the performance of the proposed weight-based missing imputation method compared to the other existing missing value imputation methods, we took into account nine widely used well-known missing imputation methods: zero imputation, mean imputation, median imputation, half of the minimum value imputation, kNN imputation, BPCA imputation, PPCA imputation, EM-PCA imputation, and RF imputation. We also considered five recently developed missing imputation techniques: GSimp, QRILC, BayesMetab, rmiMAE, and MICE. We measured the performances of the missing imputation methods, including the proposed technique, using both artificial and real data analysis in the absence and presence of different rates of outliers.

## Material and methods

In this dissertation, we developed a new missing data imputation method by minimising the two-way kernel weighted square error loss function. To compare the competence of the proposed method, we considered nine widely used traditional missing imputation techniques as described above. Substituting all missing values are by zero is known as zero imputation. In the mean, median, and half of the minimum value imputation, missing data for each metabolite are substituted by the corresponding metabolite average, median, and half of the minimum value, respectively. Missing data substitution using kNN, EM-PCA, and RF are found in the “*impute*”, “*missMDA*” and “*missForest*” packages, respectively of the R platform. Moreover, BPCA and PPCA imputation can be done using “*pcaMethods*” package in Bioconductor. As comparators of our proposed missing imputation method, we also considered five recently developed missing imputation techniques: GSimp, QRILC, BayesMetab, rmiMAE, and MICE. Among the techniques, rmiMAE is a comparatively more robust missing imputation technique which is computed by minimising the two-way mean absolute error loss function, i.e., L1 (Least absolute deviation) loss function like minimizing $\frac{1}{n}\sum\nolimits_{j = 1}^{n} {\left| {e_{ij} } \right|} = \frac{1}{n}\sum\nolimits_{j = 1}^{n} {|x_{ij} - r_{i} c_{j} } |$, which is more robust against outliers than L2 (Least square error) loss function like minimizing $\sum\nolimits_{j = 1}^{n} {(e_{ij} )^{2} } = \sum\nolimits_{j = 1}^{n} {(x_{ij} - r_{i} c_{j} } )^{2}$. To reduce the influence of outliers in the least square error loss function, here, we used the weighted squared error loss function, where the weight function is $w_{j} = \exp \left\{ { - \frac{\lambda }{{2(mad(x_{j} ))^{2} }}(x_{ij} - median(x_{j} ))^{2} } \right\}$. The speciality of the weight function is that the weight will be close to zero if the corresponding observation is apart from its median and if the corresponding observation is the neighbour of the median, the weight will be close to one. A detailed description of the proposed missing value imputation method using a two-way kernel weighted square error loss function is given below.

### Missing data imputation using two-way kernel weighted least square error approach (proposed)

Let $X = \left( {x_{ij} } \right)$ be metabolomics data, where $i = 1,2, \ldots ,p$ represents the metabolites and $j = 1,2, \ldots ,n$ represents the samples. Thus, in the metabolomics data *X,* different rows indicate different metabolites, and the columns indicate different samples.

$$X = \left( {\begin{array}{*{20}l} {x_{11} } \hfill & {x_{12} } \hfill & \cdots \hfill & {x_{1n} } \hfill \\ {x_{21} } \hfill & {x_{22} } \hfill & \cdots \hfill & {x_{2n} } \hfill \\ \vdots \hfill & \vdots \hfill & \ddots \hfill & \vdots \hfill \\ {x_{p1} } \hfill & {x_{p2} } \hfill & \cdots \hfill & {x_{pn} } \hfill \\ \end{array} } \right)$$

Each cell of the metabolomics data could be represented as the product of the metabolite (row) effect and the sample (column) effect. Mathematically, it is written in a bilinear form,

1 $$x_{ij} = r_{i} c_{j}$$

where *r*_*i*_ and *c*_*j*_ represent the *i*-th row effect (i.e. metabolite effect) and the *j*-th column effect (i.e. sample effect), respectively. The observed metabolomic data matrix usually contains missing cells and outliers. Thus, both the missing cell and outliers in the data matrix can be estimated by considering the effect of the corresponding row and column. In Eq. (1), *r*_*i*_ and *c*_*j*_ are both unknown. Therefore, our motive is to determine *r*_*i*_ and *c*_*j*_ to forecast the *ij-*th missing cell or outlying cell. To estimate *r*_*i*_ and *c*_*j*_, consider model

2 $$x_{ij} = r_{i} c_{j} + \in_{ij} ,$$

where $x_{ij}$ is the yield corresponding to the effect of the *i*th metabolite (row) and *j*th sample (column), *r*_*i*_ indicates the factors of the *i*th metabolite, and *c*_*j*_ indicates the factors of the *j-*th sample and $\in_{ij}$ indicates the error term. From model (2), we must estimate *r*_*i*_ and *c*_*j*_ simultaneously. To estimate *r*_*i*_ and *c*_*j*_, we developed a weighted least square approach using a kernel weight function $w_{j} = \exp \left\{ { - \frac{\lambda }{{2(mad(x_{j} ))^{2} }}(x_{ij} - median(x_{j} ))^{2} } \right\}$ and updated *r*_*i*_ and *c*_*j*_ by an iterative procedure, where *mad* represents the median absolute deviation. The speciality of the kernel weight function is that it lies between zero and one. The weight will be close to zero if the corresponding observation is apart from its median. If the corresponding observation is the neighbour of the median, the weight will be close to one. In the kernel weight function, $\lambda$ is the tuning parameter, where the value of $\lambda$ is chosen by k-fold cross-validation. The details of the appropriate $\lambda$ selection procedure are given in Supplementary Information 1 (Supplementary Fig. S1). If the data set is clean (i.e. no outliers), then $\lambda$ will be zero. In this condition, all the weights will be 1, that is, the technique will be the classical least-squares approach. The steps for estimating *r*_*i*_ and *c*_*j*_ are given below:

**Step 1**: To initialise the *j*-th column (sample) effect (*c*_*j*_), calculate the *j-*th column median of *X*. Column median is computed by excluding the missing values $j = 1,2, \ldots ,n$.
**Step 2**: Using the weighted least square approach, estimate the *i-*th row effect (i.e. metabolite effect) *r*_*i*_ by minimising $\sum\nolimits_{j = 1}^{n} {\left( {e_{ij} } \right)^{2} } = \sum\nolimits_{j = 1}^{n} {w_{ij} \left( {x_{ij} - r_{i} c_{j} } \right)^{2} }$, based on the *i-*th row of *X*, by eliminating the missing values, $i = 1,2, \ldots ,p$.
**Step 3**: Revise the *j-*th column effect $c_{j}$, using the weighted least square approach by minimising $\sum\nolimits_{i = 1}^{p} {w_{ij} \left( {x_{ij} - r_{i} c_{j} } \right)^{2} }$, based on the *j-*th column of *X*, by eliminating the missing values, $j = 1,2, \ldots ,n$.
**Step 4**: Repeat Steps 2 and 3 until it satisfies the rule $\frac{{|{\varvec{r}}_{{{{new}}}} - {\varvec{r}}_{{{{old}}}} | + |{\varvec{c}}_{{{{new}}}} - {\varvec{c}}_{{{{old}}}} |}}{n + p} \le \varepsilon$; here $\varepsilon$ is a very small positive number, which depends on the researcher’s interest. Here, we choose $\varepsilon = 0.01$.
**Step 5**
  - Compute the first fitted bilinear form as $\hat{X}^{(1)} = {\hat{\varvec{r}}}_{1} {\hat{\varvec{c}}}_{1}$, where ${\hat{\varvec{r}}}_{1} = (\hat{r}_{1} ,\hat{r}_{2} , \ldots ,\hat{r}_{p} )^{T}$ and ${\hat{\varvec{c}}}_{1} = (\hat{c}_{1} ,\hat{c}_{2} , \ldots ,\hat{c}_{n} )$ are obtained from Step 4.
  - Calculate the first remainder matrix (*X*_*R1*_) as $X_{R1} = X - \hat{X}^{(1)} = X - {\hat{\varvec{r}}}_{1} {\hat{\varvec{c}}}_{1}$ (excluding the missing cells of the data matrix)
  - Using steps 1–4 on *X*_*R1*_, compute the second fitted bilinear form as,$\hat{X}_{R1} = {\hat{\varvec{r}}}_{2} {\hat{\varvec{c}}}_{2}$ and calculate the second remainder matrix (*X*_*R*2_) as, $X_{R2} = X_{R1} - \hat{X}_{R1} = X - {\hat{\varvec{r}}}_{1} {\hat{\varvec{c}}}_{1} - {\hat{\varvec{r}}}_{2} {\hat{\varvec{c}}}_{2}$ (excluding the missing cells of the data matrix)
  - Similarly, calculate the *r*-th remainder (*X*_*Rr*_) as, $X_{Rr} = X_{R(r - 1)} - \hat{X}_{R(r - 1)} = X - \sum\nolimits_{k = 1}^{r} {{\hat{\varvec{r}}}_{k} {\hat{\varvec{c}}}_{k} }$ that is, $X = X_{Rr} + \sum\nolimits_{k = 1}^{r} {{\hat{\varvec{r}}}_{k} {\hat{\varvec{c}}}_{k} }$. The number of *r* is selected in such a way that the total row variations of $\sum\nolimits_{k = 1}^{r} {{\hat{\varvec{r}}}_{k} {\hat{\varvec{c}}}_{k} }$ can explain (1 − *α*)100*%* variations of *X* (using the concept of singular value decomposition; the details of the *r* selection procedure are given in Appendix 1 of the supplementary materials), where *α* is chosen by the researcher interest. In this case, *α* = 0.05. Therefore, the approximation of *X* is:
    3 $$X \approx \hat{X}^{(r)} = \sum\limits_{k = 1}^{r} {{\hat{\varvec{r}}}_{k} {\hat{\varvec{c}}}_{k} }$$
**Step 6**: Substitute the missing values and the outlying cells of *X* by the corresponding cells of $\hat{X}^{(r)}$ that produce the reconstructed full and clean data matrix $\tilde{X}$. Here, the inter quartile range (IQR) rule^31^ was used to detect outliers.

The application procedure of the proposed method in metabolomics data is given below. The metabolomics dataset may contain several groups of samples in their data structure. If a metabolomics dataset contains *k* groups of samples, then the dataset is split according to the groups as

$$X = \left[ {\overbrace {{\begin{array}{*{20}l} {x_{11} } \hfill & {x_{12} } \hfill & \cdots \hfill & {x_{{1g_{1} }} } \hfill \\ {x_{21} } \hfill & {x_{22} } \hfill & \cdots \hfill & {x_{{2g_{1} }} } \hfill \\ \vdots \hfill & \vdots \hfill & \ddots \hfill & \vdots \hfill \\ {x_{p1} } \hfill & {x_{p2} } \hfill & \cdots \hfill & {x_{{pg_{1} }} } \hfill \\ \end{array} }}^{group - 1}\overbrace {{\begin{array}{*{20}l} {x_{{1(g_{1} + 1)}} } \hfill & {x_{{1(g_{1} + 2)}} } \hfill & \cdots \hfill & {x_{{1(g_{1} + g_{2} )}} } \hfill \\ {x_{{2(g_{1} + 1)}} } \hfill & {x_{{2(g_{1} + 2)}} } \hfill & \cdots \hfill & {x_{{2(g_{1} + g_{2} )}} } \hfill \\ \vdots \hfill & \vdots \hfill & \ddots \hfill & \vdots \hfill \\ {x_{{p(g_{1} + 1)}} } \hfill & {x_{{p(g_{1} + 2)}} } \hfill & \cdots \hfill & {x_{{p(g_{1} + g_{2} )}} } \hfill \\ \end{array} }}^{group - 2}\overbrace {{\begin{array}{*{20}l} \cdots \hfill \\ \cdots \hfill \\ \ddots \hfill \\ \cdots \hfill \\ \end{array} }}^{ \cdots }\overbrace {{\begin{array}{*{20}l} {x_{{1(g_{1} + \cdots + g_{k - 1} + 1)}} } \hfill & {x_{{1(g_{1} + \cdots + g_{k - 1} + 2)}} } \hfill & \cdots \hfill & {x_{{1(g_{1} + \cdots + g_{k} )}} } \hfill \\ {x_{{2(g_{1} + \cdots + g_{k - 1} + 1)}} } \hfill & {x_{{2(g_{1} + \cdots + g_{k - 1} + 2)}} } \hfill & \cdots \hfill & {x_{{2(g_{1} + \cdots + g_{k} )}} } \hfill \\ \vdots \hfill & \vdots \hfill & \ddots \hfill & \vdots \hfill \\ {x_{{p(g_{1} + \cdots + g_{k - 1} + 1)}} } \hfill & {x_{{p(g_{1} + \cdots + g_{k - 1} + 2)}} } \hfill & \cdots \hfill & {x_{{p(g_{1} + \cdots + g_{k} )}} } \hfill \\ \end{array} }}^{group - k}} \right]$$

where $g_{1}$ is the column number (subjects) of group-1, $g_{2}$ is the column number (subjects) of group-2, and so on $g_{1} + g_{2} + \cdots + g_{k} = n$.

Therefore, we checked whether the metabolomics data matrix *X* contained multiple groups in the samples. If *X* contains multiple groups, then partition matrix *X* as $$X = (\begin{array}{*{20}c} {X_{1} } & {X_{2} } & \cdots & {X_{k} } \\ \end{array} )$$ according to *k* groups of samples,

$$\begin{aligned} & {\text{where}}\;\;\;X_{1} = \left( {\begin{array}{*{20}l} {x_{11} } \hfill & {x_{12} } \hfill & \cdots \hfill & {x_{{1g_{1} }} } \hfill \\ {x_{21} } \hfill & {x_{22} } \hfill & \cdots \hfill & {x_{{2g_{1} }} } \hfill \\ \vdots \hfill & \vdots \hfill & \ddots \hfill & \vdots \hfill \\ {x_{p1} } \hfill & {x_{p2} } \hfill & \cdots \hfill & {x_{{pg_{1} }} } \hfill \\ \end{array} } \right),\;\;X_{2} = \left( {\begin{array}{*{20}l} {x_{{1(g_{1} + 1)}} } \hfill & {x_{{1(g_{1} + 2)}} } \hfill & \cdots \hfill & {x_{{1(g_{1} + g_{2} )}} } \hfill \\ {x_{{2(g_{1} + 1)}} } \hfill & {x_{{2(g_{1} + 2)}} } \hfill & \cdots \hfill & {x_{{2(g_{1} + g_{2} )}} } \hfill \\ \vdots \hfill & \vdots \hfill & \ddots \hfill & \vdots \hfill \\ {x_{{p(g_{1} + 1)}} } \hfill & {x_{{p(g_{1} + 2)}} } \hfill & \cdots \hfill & {x_{{p(g_{1} + g_{2} )}} } \hfill \\ \end{array} } \right)\;\;{\text{and}} \\ & X_{k} = \left( {\begin{array}{*{20}l} {x_{{1(g_{1} + \cdots + g_{k - 1} + 1)}} } \hfill & {x_{{1(g_{1} + \cdots + g_{k - 1} + 2)}} } \hfill & \cdots \hfill & {x_{{1(g_{1} + \cdots + g_{k} )}} } \hfill \\ {x_{{2(g_{1} + \cdots + g_{k - 1} + 1)}} } \hfill & {x_{{2(g_{1} + \cdots + g_{k - 1} + 2)}} } \hfill & \cdots \hfill & {x_{{2(g_{1} + \cdots + g_{k} )}} } \hfill \\ \vdots \hfill & \vdots \hfill & \ddots \hfill & \vdots \hfill \\ {x_{{p(g_{1} + \cdots + g_{k - 1} + 1)}} } \hfill & {x_{{p(g_{1} + \cdots + g_{k - 1} + 2)}} } \hfill & \cdots \hfill & {x_{{p(g_{1} + \cdots + g_{k} )}} } \hfill \\ \end{array} } \right); \\ & {\text{otherwise}},\;\;X = \left( {\begin{array}{*{20}l} {x_{11} } \hfill & {x_{12} } \hfill & \cdots \hfill & {x_{1n} } \hfill \\ {x_{21} } \hfill & {x_{22} } \hfill & \cdots \hfill & {x_{2n} } \hfill \\ \vdots \hfill & \vdots \hfill & \ddots \hfill & \vdots \hfill \\ {x_{p1} } \hfill & {x_{p2} } \hfill & \cdots \hfill & {x_{pn} } \hfill \\ \end{array} } \right) \\ \end{aligned}$$

If *X* contains *k* groups, then apply Steps 1–6 for each partitioned data matrix and compute $$\begin{array}{*{20}c} {\tilde{X}_{1} ,} & {\tilde{X}_{2} ,} & \cdots & {\tilde{X}_{k} } \\ \end{array}$$. Thus, the reconstructed full and clean data matrix $$\tilde{X} = \begin{array}{*{20}c} {(\tilde{X}_{1} } & {\tilde{X}_{2} } & \cdots & {\tilde{X}_{k} } \\ \end{array} )$$. Otherwise, apply Steps 1–6 for the data matrix *X* and compute the reconstructed full and clean data matrix $\tilde{X}$.

User can install the package in R platform using the following R code

- library(devtools)
- install_github("NishithPaul/tWLSA")
- library(tWLSA)

### Artificially generated metabolomics data

To simulate metabolomics datasets, we used the following additive linear model:

4 $$x_{ijk} = \mu_{i} + g_{ij} + \in_{ijk}$$

where $x_{ijk}$ is the concentration of the *i*^th^ metabolite, *j*^th^ group, and *k*^th^ sample; the average concentration for the *i*-th metabolite is $\mu_{i}$; $g_{ij}$ represents the *j*^th^ group effect of the *i*^th^ metabolite and the random error term of the *i*-th metabolite, *j*-th group, and *k*-th sample is $\in_{ijk}$. To generate the data, we considered, $\mu_{i} \sim uniform(5,10)$ and $\in_{ijk} \sim N(0,1)$. To measure the efficiency of the proposed technique, we created three types of metabolomics datasets: (i) without a class level in the samples, (ii) two class levels (two groups) in the samples, and (iii) three class levels (three groups) in the samples. In the case of two-and three-class level-based datasets, we also generated two types of metabolites: (a) equal concentration (EE) metabolites and (b) differential concentrations (DE) metabolites. DE metabolites were classified into two groups: upregulated and down-regulated metabolites. For up-concentrated metabolites, we used $g_{ij} \sim N(0,1)$ the healthy group and $g_{ij} \sim N(2,1)$ the disease group. Similarly, for down-regulated metabolites, we used $g_{ij} \sim N(2,1)$ the healthy group and $g_{ij} \sim N(0,1)$ the disease group. For EE metabolites, $g_{ij} \sim N(0,1)$ in both groups. We generated 200 metabolites and 90 samples for each dataset. In two-and three-class datasets, we considered 80 metabolites as DE and 120 metabolites as EE. We generated 100 datasets for each type of dataset. We also incorporated various rates (5%, 10%, 15%, and 20%) of missing cells in the data matrix. Among the total missing values, 60% MAR and 40% for lower values. To investigate the efficiency of our proposed technique in the presence of outliers, we also included various rates (3%, 5%, 7%, and 10%) of outliers in the artificial datasets. In the *i*-th metabolite, we provided *N*(5*μ_i_, σ_i_^2^) *as* outliers, where *μ*_*i*_ and *σ*_*i*_^2^ are the mean and variance of the i-th metabolite; these outliers were distributed randomly in the dataset; thus, outliers may occur anywhere in the dataset.

### Real metabolomics data

To measure the performance of our proposed missing imputation method, we first considered two publicly available fully defined real metabolomics data matrices. One is the Human Cachexia dataset^32^, collected from ^1H^-NMR profiles of urinary metabolites that are available in the R-specmine library. The other is the treated dataset^33^, which is also available in the R-metabolomics library. Since, these two data matrices did not contain any missing values, to investigate the efficiency of the proposed technique compared to the other techniques we randomly incorporated different rates (5%, 10%, 15% and 20%) of missing values and also computed the mean square error (MSE) between the reconstructed data and original data. We also considered two datasets: hepatocellular carcinoma (HCC) with 26.52% missing values/cells^34^ and MDA-MB-231 breast cancer dataset with 15.81% missing values^35^ to evaluate the performance of the proposed missing value imputation method. The HCC and MDA-MB-231 datasets were also modified by artificially including various rates (3%, 5%, 7%, and 10%) of outliers to investigate the performance of the proposed method. Outliers are distributed randomly and follow *N*(5*μ_i_, σ_i_^2^), where *μ*_*i*_ and *σ*_*i*_^2^ are the mean and variance of the i-th metabolite, respectively.

## Results

To demonstrate the performance of the proposed missing imputation technique compared to the extensively used conventional techniques and recently developed missing imputation techniques, we analysed both artificial and experimentally measured metabolomics datasets.

### Artificial data analysis results

**Figure 1:**
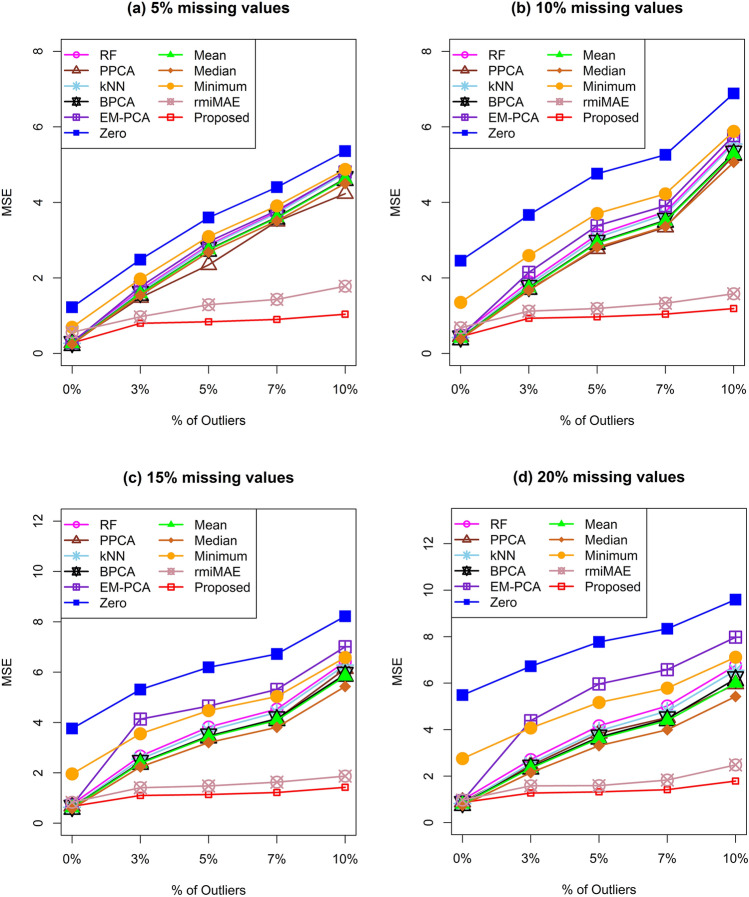
Performance investigation of different missing imputation techniques using average MSE for without class level data.

In simulation studies, we first measured the performance of the proposed missing imputation technique compared to the other ten missing imputation methods (zero, mean, median, half of the minimum value, kNN, BPCA, PPCA, EM-PCA, RF imputations and rmiMAE) using the distance-based measurement. We computed the MSE between the original simulated dataset and the reconstructed missing imputed dataset in both the presence and absence of outliers. We generated three types of simulated metabolomics datasets and 100 datasets for each type and calculated the average MSE from 100 MSEs for each type of dataset for different rates of outliers (0%, 3%, 5%, 7%, and 10%) and different rates (5%, 10%, 15%, and 20%) of missing values. For the datasets with no class level in the samples, the results of the above calculation are shown in Fig. 1. Similarly, for two class levels (two groups) in the sample datasets and three class levels (three groups) in the sample datasets, the results of the aforementioned calculation are given in the Supplementary Information in Fig. S1 and Fig. S2. In the same way, a comparison of the performance of our proposed method with the recently developed techniques (GSimp, QRILC, BayesMetab, rmiMAE, and MICE) using the datasets with no class level in the samples are given in the Supplementary Information in Fig. S3. In all these figures, the proposed missing value imputation technique produced lower average MSEs for various rates (0%, 3%, 5%, 7%, and 10%) of outliers, as well as for various rates (5%, 10%, 15%, and 20%) of missing values. Therefore, our missing imputation method was better than the other existing techniques.

Second, we evaluated the performance of our developed KMI method using the misclassification error rate (MER), receiver operating characteristic (ROC) curve, and area under the ROC curve (AUC) through DE metabolite identification for two groups and three groups of datasets. To calculate the performance indices (MER, ROC curve, and AUC values), we identified the DE metabolites from the different reconstructed datasets (missing were imputed by different methods) using *a t*-test for the two class level dataset and analysis of variance (ANOVA) for the multiclass level dataset. Since the DE and EE metabolites were known in the simulated dataset, we computed the MER, ROC curve, and AUC for different missing imputed datasets in both the absence and presence of various rates of outliers. The above calculation procedures are provided in Supplementary Information in Fig. S4.

**Figure 2:**
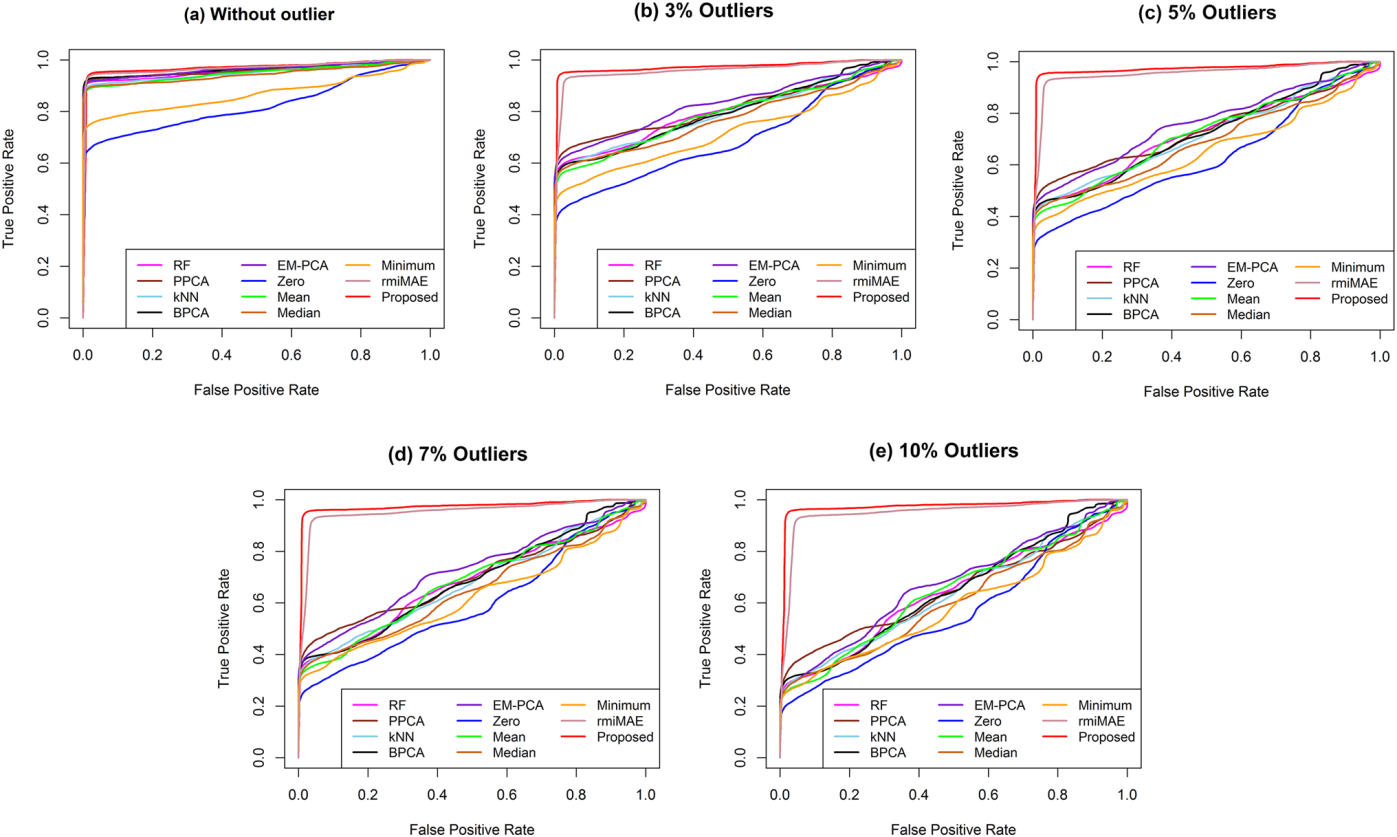
Performance investigation of different missing value imputation techniques using receiver operating characteristic curve of DE calculation for two class level dataset with 5% missing values in absence and presence of outliers.

The ROC curve of DE calculation for two-class datasets with 5% missing data and various rates of outliers are depicted in Fig. 2. Similarly, for three classes of simulated datasets, the ROC curve of the DE calculation is also shown in the Supplementary Information in Fig. S5. Similarly, for 10%, 15%, and 20% missing values, the ROC curves are given in the Supplementary Information (Fig. S6–S11). In addition, Table 1 presents the MER and AUC values of the DE calculation for two-class datasets with 5% missing as well as various rates of outliers. Moreover, for the two classes of datasets with 5% missing as well as various rates of outliers, the MER and AUC values of DE identification are also presented in the Supplementary Information in Table S1. Similarly, for 10%, 15%, and 20% missing values, the MER and AUC values of the DE calculation are also given in the Supplementary Information (Tables S2 to S7). The results of the performance measures of Fig. 2, Table 1, Fig. S5 to S11, and Tables S1 to S7 show that the proposed missing imputation method produced lower average MER and higher average AUC values for different rates (5%, 10%, 15%, and 20%) of missing values and various rates (0%, 3%, 5%, 7%, and 10%) of outliers. Therefore, the proposed KMI technique was better than the existing missing value imputation techniques.

**Table 1:** Average misclassification error rate (MER) and area under the receiver operating characteristic curve (AUC) of DE calculation for two class simulated data with 5% missing values and different rates of outliers.

Methods	Without outliers MER (AUC)	3% outliers MER (AUC)	5% outliers MER (AUC)	7% outliers MER (AUC)	10% outliers MER (AUC)
RF	4.23 (0.956)	19.76 (0.797)	27.41 (0.720)	31.39 (0.679)	35.52 (0.638)
PPCA	3.77 (0.964)	18.59 (0.811)	26.03 (0.737)	30.06 (0.698)	34.48 (0.652)
kNN	4.83 (0.952)	20.41 (0.794)	27.85 (0.719)	31.83 (0.678)	35.93 (0.637)
BPCA	3.45 (0.967)	21.31 (0.788)	28.65 (0.703)	32.34 (0.675)	36.45 (0.641)
EM-PCA	3.38 (0.969)	18.76 (0.829)	26.08 (0.733)	30.09 (0.707)	36.03 (0.649)
Zero	16.57 (0.829)	28.88 (0.709)	34.15 (0.651)	37.13 (0.623)	39.95 (0.598)
Mean	5.23 (0.949)	21.29 (0.789)	28.62 (0.719)	32.37 (0.672)	36.41 (0.642)
Median	5.12 (0.951)	20.99 (0.791)	28.36 (0.706)	32.18 (0.676)	36.25 (0.643)
Minimum	12.44 (0.867)	26.45 (0.728)	32.26 (0.673)	35.52 (0.640)	38.75 (0.604)
rmiMAE	2.94 (0.971)	4.21 (0.958)	4.77 (0.951)	4.98 (0.948)	5.13 (0.964)
Proposed	**2.73 (0.973)**	**2.93 (0.971)**	**2.98 (0.970)**	**3.03 (0.969)**	**3.05 (0.969)**

Bold indicates the lower MER and Higher AUC throughout the column.

**Figure 3:**
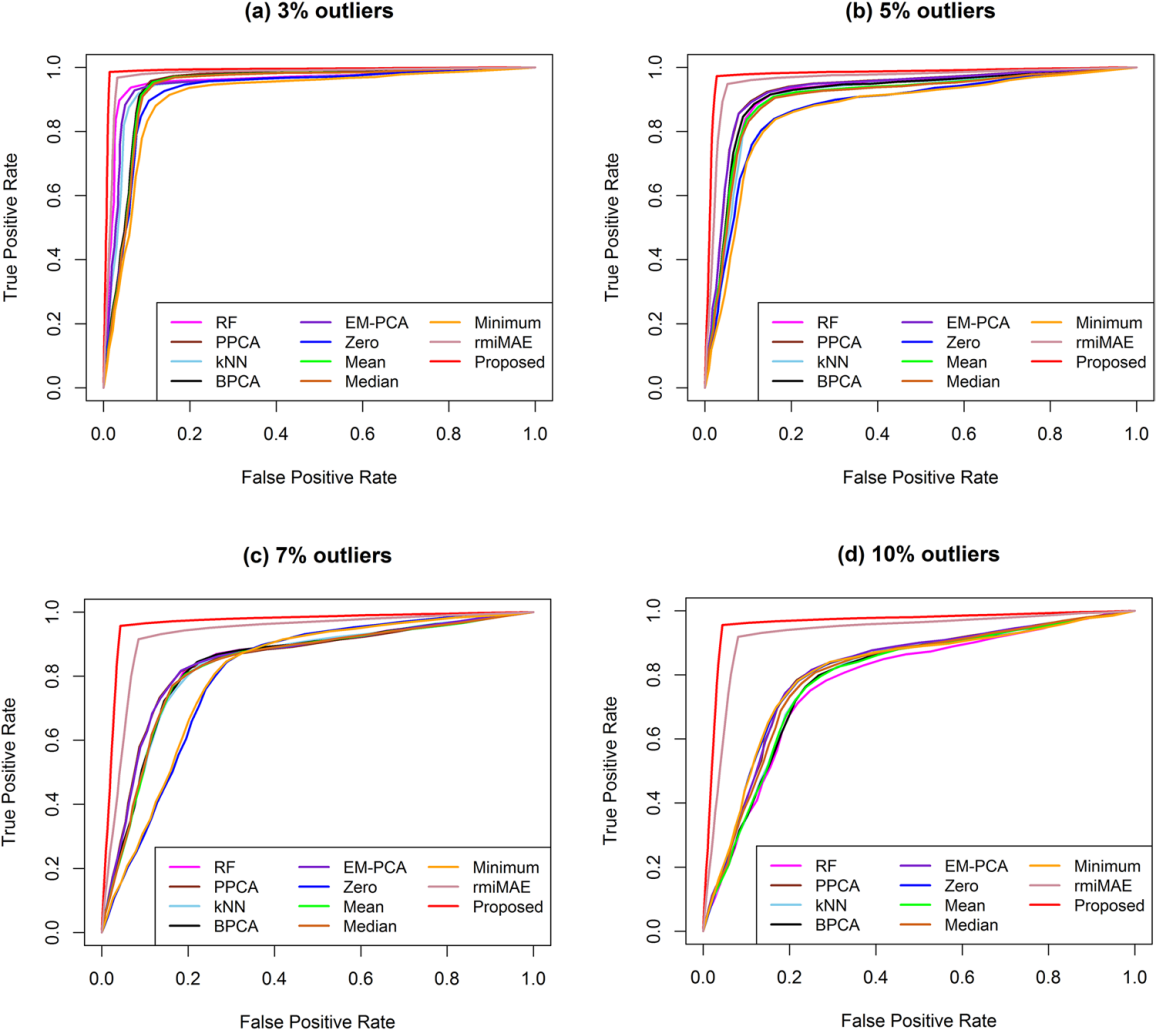
Performance investigation of different missing value imputation techniques using receiver operating characteristic curve of sample classification for two class level dataset with 5% missing values in presence of outliers.

Finally, we measured the performance of our proposed KMI technique through sample classification using only DE metabolites. Although taking only the differentially expressed variables may give over-optimistic values for prediction performance, however, to increase accuracy, it is often used as the feature selection approach. To overcome this problem we used the cross-validation approach. The performance measure calculation procedure of different imputation methods based on sample classification (using a SVM classifier) is given in the Supplementary Information in Fig. S12. The ROC curve based on sample classification using a test dataset for two-class simulated datasets with 5% and 10% missing values and various rates (3%, 5%, 7%, and 10%) of outliers are presented in Fig. 3 and Supplementary Fig. S13, respectively. Figure 3 and Fig. S13 show that our proposed KMI technique gave a higher average true positive rate at any point of average false positive rate compared to the other missing imputation methods in the presence of different rates of outliers (3%, 5%, 7%, and 10%). We also computed the average MER and AUC in the appearance of 5% missing data as well as the different percentages of outliers using two-and three class level datasets, which are presented in Tables 2 and 3. Similarly, for 10% missing data, as well as different percentages of outliers using two-and three class level datasets, the average MER and AUC are given in Supplementary Tables S8 and S9. Tables 2 and 3 show that the proposed KMI technique produced lower average MER and higher average AUC values at various rates of missing values and different rates of outliers for two-and three class level simulated metabolomics data. Therefore, in simulation studies, our proposed KMI technique was better than the existing missing value imputation methods.

**Table 2:** Average misclassification error rate(MER) and area under the receiver operating characteristic curve (AUC) for two class simulated data with 5% missing values and different rates of outliers.

Methods	3% Outliers MER (AUC)	5% Outliers MER (AUC)	7% Outliers MER (AUC)	10% Outliers MER (AUC)
RF	4.10 (0.9516)	8.67 (0.9143)	16.17 (0.8395)	17 (0.8332)
PPCA	5.67 (0.9408)	7.37 (0.9276)	15.53 (0.8454)	19.37 (0.8048)
kNN	5.53 (0.9412)	9.27 (0.9054)	16.57 (0.83885)	19.6 (0.8042)
BPCA	5.77 (0.9391)	8.50 (0.9149)	16.27 (0.84025)	21.2 (0.7882)
EM-PCA	5.03 (0.9460)	7.43 (0.9270)	15.33 (0.8475)	15.77 (0.8385)
Zero	7.73 (0.9224)	12.70 (0.8759)	19 (0.8068)	18.9 (0.8114)
Mean	5.93 (0.9371)	9.30 (0.9059)	16.5 (0.8358)	21.37 (0.7858)
Median	6.17 (0.9353)	9.67 (0.9021)	16.43 (0.8366)	19.87 (0.8021)
Minimum	8.67 (0.9088)	13.70 (0.8675)	18.87 (0.8088)	19.27 (0.8073)
rmiMAE	1.69 (0.9831)	1.81 (0.9819)	2.36 (0.9764)	2.82 ( 0.9718)
Proposed	**1.27 (0.9882)**	**1.30 (0.9878)**	**1.30 (0.9876)**	**1.32 (0.9872)**

Bold indicates the lower MER and Higher AUC throughout the column.

**Table 3:** Average misclassification error rate and area under the receiver operating characteristic curve (AUC) for three class simulated data with 5% missing values and different rates of outliers.

Methods	3% Outliers MER (AUC)	5% Outliers MER (AUC)	7% Outliers MER (AUC)	10% Outliers MER (AUC)
RF	5.60 (0.9661)	10.60 (0.9122)	12.97 (0.8631)	21.63 (0.7687)
PPCA	4.33 (0.9718)	7.73 (0.9375)	11.03 (0.8797)	21.47 (0.7734)
kNN	3.67 (0.9783)	9.40 (0.9251)	13.80 (0.8634)	18.60 (0.7785)
BPCA	4.00 (0.9735)	10.17 (0.9131)	13.37 (0.8688)	21.77 (0.7562)
EM-PCA	5.20 (0.9645)	8.93 (0.9302)	11.07 (0.8799)	21.67 (0.7674)
Zero	4.67 (0.9696)	8.67 (0.9172)	15.80 (0.8546)	15.20 (0.8054)
Mean	4.13 (0.9724)	10.97 (0.8995)	13.93 (0.8620)	18.23 (0.7924)
Median	4.20 (0.9721)	10.03 (0.9152)	13.50 (0.8673)	17.93 (0.7948)
Minimum	5.23 (0.9626)	8.30 (0.9193)	12.13 (0.8913)	14.33 (0.8214)
rmiMAE	1.82 (0.9816)	2.21 (0.9783)	2.57 (0.9721)	3.29 (0.9672)
Proposed	**1.28 (0.9892)**	**1.32 (0.9877)**	**1.39 (0.9862)**	**1.48 (0.9835)**

Bold indicates the lower MER and Higher AUC throughout the column.

### Real data analysis results

**Figure 4:**
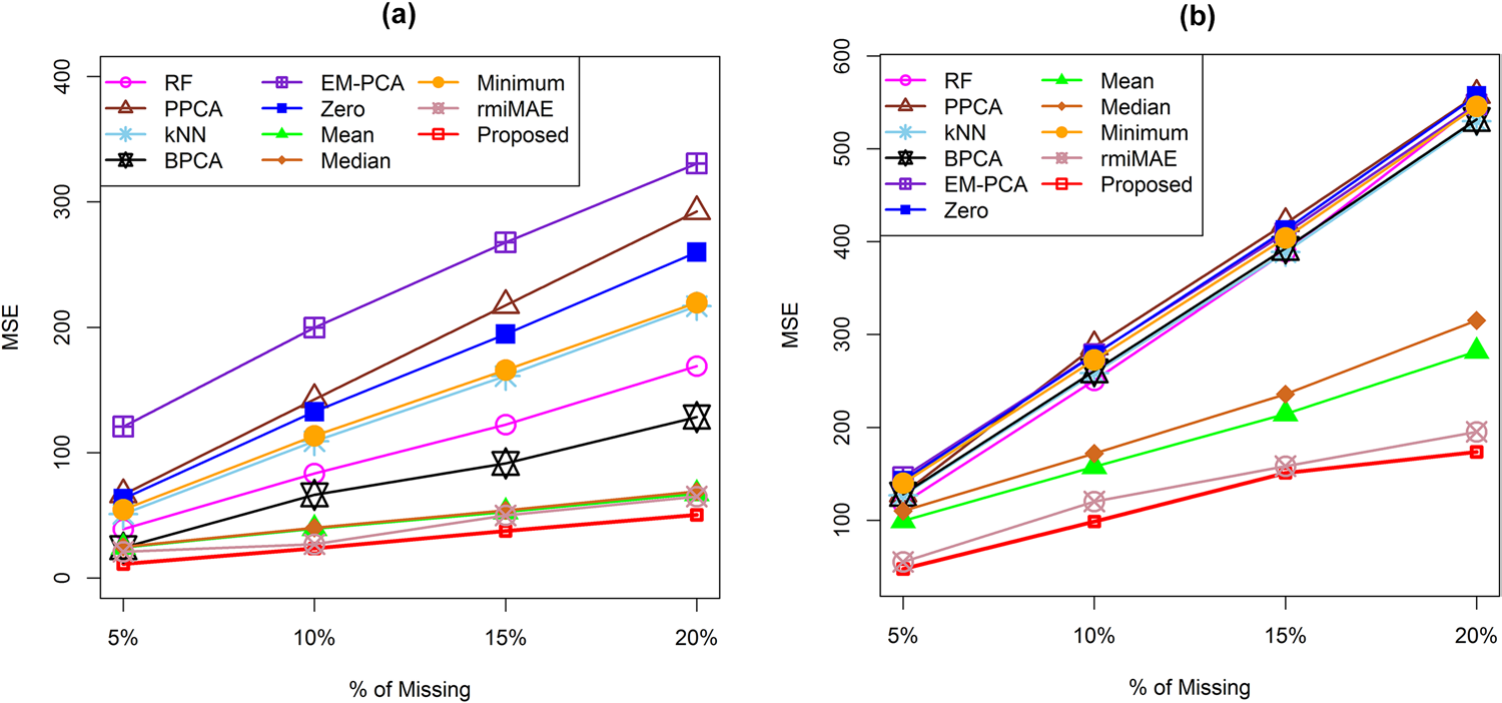
Performance investigation of different missing value imputation techniques using MSE calculation for different rates of missing values of (**a**) Human Cachexia dataset and (**b**) treated dataset.

Here, we used four real metabolomics datasets to evaluate the efficiency of our newly developed KMI technique compared to other missing imputation methods for real data analysis. Since the Human Cachexia and treated datasets are fully defined, to explore the performance of our proposed technique we artificially incorporated various percentage of missing values (5%, 10%, 15% and 20%) and reconstructed the data matrix using several missing value imputation methods including the proposed one. We measured the MSE between the original and reconstructed datasets. We also repeated the aforementioned calculation 100 times and computed the average MSE for different rates of missing values, as presented in Fig. 4. The figure shows that the proposed missing value imputation technique produced a lower average MSE for different rates of missing values for the Human Cachexia dataset (Fig. 4a) and the treated dataset (Fig. 4b). Therefore, our proposed imputation method displayed comparatively better performance than the other ten conventional missing value imputation methods. Moreover, we conducted a comparative study of the efficiency of our proposed missing imputation technique and five recently developed techniques (GSimp, QRILC, BayesMetab, rmiMAE, and MICE) using MSE on the Cachexia dataset with various rates of missing values. This is presented in the Supplementary Information in Fig. S14.

**Figure 5:**
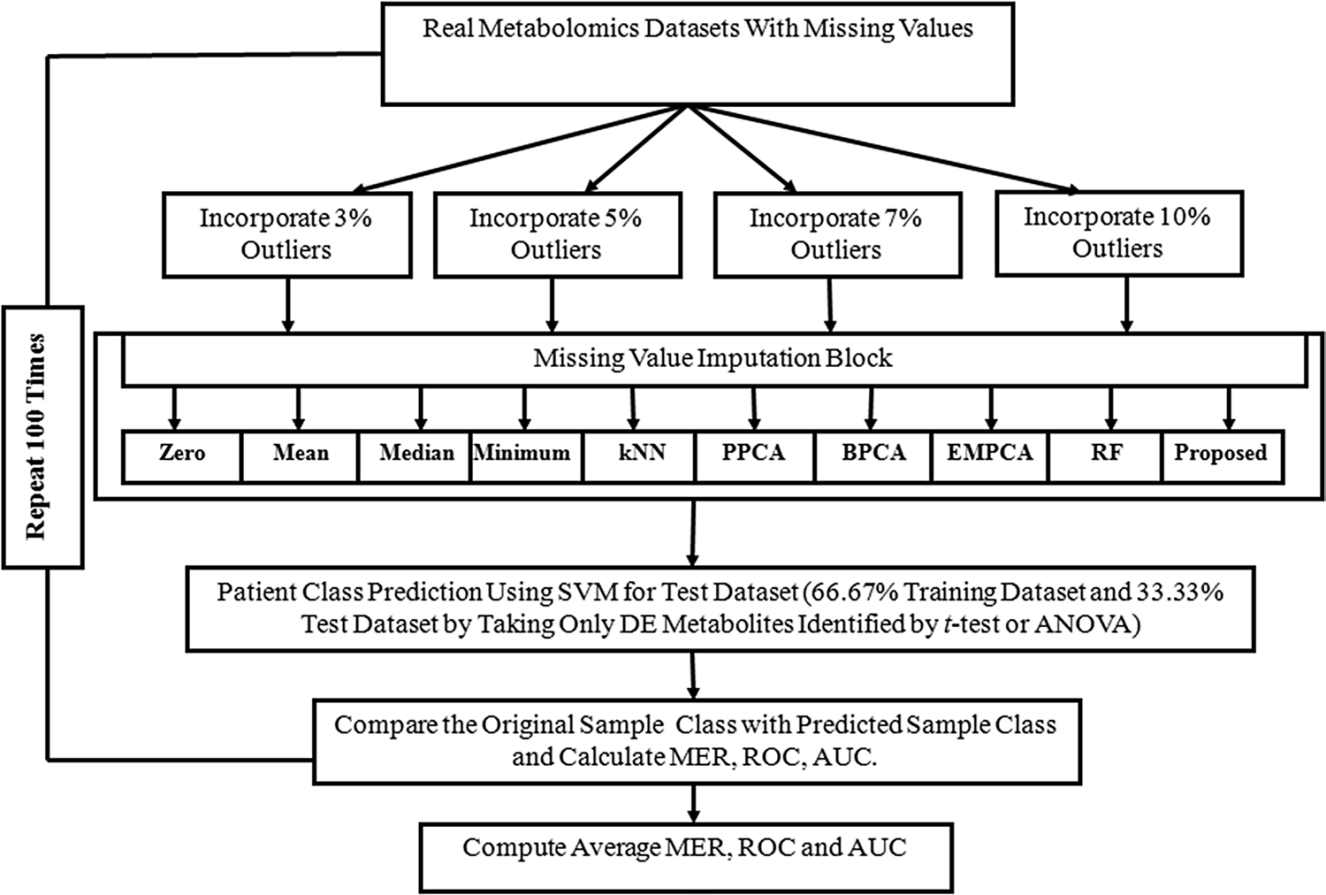
Performance measures calculation procedure for real dataset on the basis of sample classification.

We also measured the competency of our proposed KMI technique using MER and AUC of sample classification for both the two-class hepatocellular carcinoma dataset and the three-class MDA-MB-231 dataset. To evaluate the performance of all well-known missing value imputation methods in the presence of outliers, we modified both datasets by artificially incorporating different rates of outliers (3%, 5%, 7%, and 10%). The performance measure calculation procedure for different missing imputation techniques is shown in Fig. 5. The calculation of performance measures (MER and AUC) using the HCC dataset and the MDA-MB-231 dataset are shown in Tables 4 and 5, respectively. The data indicated that our proposed KMI technique produced a lower average MER and higher AUC values compared to other missing imputation methods in the appearance of various rates of outliers. Therefore, both simulation studies and real data analysis showed that our proposed missing value imputation method performed better than the existing missing value imputation methods.

**Table 4:** Average misclassification error rate and area under the receiver operating characteristic curve (AUC) of sample classification for two class real dataset (hepatocellular carcinoma) with 26.52% missing values and artificially imputed different rates of outliers.

Methods	Without outliers MER (AUC)	3% outliers MER (AUC)	5% outliers MER (AUC)	7% outliers MER (AUC)	10% outliers MER (AUC)
RF	10.67 (0.8903)	13.61 (0.8642)	20.16 (0.8001)	21.18 (0.7883)	22.61 (0.7751)
PPCA	15.22 (0.8495)	16.45 (0.8365)	26.44 (0.7364)	26.56 (0.7355)	26.67 (0.7323)
kNN	13.53 (0.8795)	13.94 (0.8676)	25.56 (0.7484)	25.97 (0.7402)	26.33 (0.7375)
BPCA	14.61 (0.8571)	16.28 (0.8342)	21.67 (0.7882)	23.38 (0.7679)	25.45 (0.7452)
EM-PCA	11.44 (0.8894)	11.58 (0.8813)	23.72 (0.7665)	23.70 (0.7648)	23.69 (0.7618)
Zero	11.55 (0.8874)	12.39 (0.8747)	15.58 (0.8433)	17.51 (0.8246)	19.44 (0.8026)
Mean	10.56 (0.8914)	13.61 (0.8644)	20.73 (0.7947)	22.69 (0.7723)	24.89 (0.7502)
Median	9.94 (0.9068)	12.38 (0.8783)	17.61 (0.8276)	20.17 (0.7975)	23.54 (0.7637)
Minimum	10.56 (0.8937)	11.24 (0.8894)	13.57 (0.8646)	15.67 (0.8427)	17.44 (0.8265)
rmiMAE	0.78 (0.9922)	1.84 (0.9815)	2.36 (0.9773)	2.97 (0.9701)	3.65 (0.9644)
Proposed	0.00 (1.00)	**0.32 (0.9972)**	**0.75 (0.9929)**	**1.18 (0.9895)**	**1.87 (0.9837)**

Bold indicates the lower MER and Higher AUC throughout the column.

**Table 5:** Average misclassification error rate and area under the receiver operating characteristic curve (AUC) of sample classification for three class real dataset (MDA-MB-231) with 15.81% missing values and artificially imputed different rates of outliers.

Methods	Without outliers MER (AUC)	3% outliers MER (AUC)	5% outliers MER (AUC)	7% outliers MER (AUC)	10% outliers MER (AUC)
RF	4.23 (0.9635)	21.57 (0.7996)	23.53 (0.7771)	31.96 (0.6951)	34.70 (0.6777)
PPCA	4.33 (0.9629)	18.27 (0.8288)	25.13 (0.7564)	21.83 (0.7938)	30.63 (0.7238)
kNN	3.43 (0.9759)	18.63 (0.8296)	21.80 (0.7940)	24.56 (0.7646)	43.56 (0.6672)
BPCA	8.07 (0.9267)	18.77 (0.8207)	22.06 (0.7836)	26.43 (0.7475)	33.70 (0.6858)
EM-PCA	4.46 (0.9619)	19.76 (0.8101)	19.63 (0.8161)	21.66 (0.7938)	34.93 (0.6741)
Zero	3.45 (0.9755)	12.20 (0.8843)	25.86 (0.7554)	27.40 (0.7347)	38.53 (0.6374)
Mean	3.73 (0.9728)	11.73 (0.8858)	25.30 (0.7597)	26.2 (0.7452)	35.90 (0.6668)
Median	3.67 (0.9732)	11.36 (0.8861)	22.73 (0.7874)	23.16 (0.7753)	34.90 (0.6748)
Minimum	3.43 (0.9758)	13.10 (0.8774)	24.64 (0.7647)	27.16 (0.7457)	37.10 (0.6457)
rmiMAE	1.45 (0.9854)	2.47 (0.9757)	3.04 (0.9753)	3.56 (0.9668)	4.01 (0.9611)
Proposed	**0.17 (0.9992)**	**0.25 (0.9983)**	**0.30 (0.9975)**	**0.52 (0.9951)**	**1.13 (0.9892)**

Bold indicates the lower MER and Higher AUC throughout the column.

## Discussion

We examined the performance of each missing imputation technique by optimising the parameter settings using a trial-and-error basis to avoid biased comparisons. For example, in the case of kNN imputation, we chose *k*, for which the MSE and MER were smaller and the accuracy was maximum. The performance of different missing imputation techniques may depend on the structure and the value/intensity of data. Therefore, we presently generated three types of simulated metabolomics datasets and 100 datasets for each type and calculated the average MSE from 100 MSEs for each type of dataset at different rates of outliers (0%, 3%, 5%, 7%, and 10%) and different rates (5%, 10%, 15%, and 20%) of missing values.

MAR may occur at any position in the data matrix, thus, we generated 100 modified real datasets, including different MAR positions of the data matrix, to measure the performance of different missing imputation techniques. To compute the performance of various missing imputation methods through MER and AUC using the classification technique, we divided the dataset into two parts: the test dataset and the training dataset. To reduce the sampling error during the calculation of MER and AUC, we generated 100 training datasets and 100 test datasets for each case and computed the average MER and AUC for measuring the performance of different missing imputation methods. The detailed calculation procedure of different performance measures calculated by different missing imputation methods for the artificial dataset is shown in the Supplementary Information in Fig. S4 and Fig. S12. As well, information for the artificial dataset is presented in Fig. 5. We calculated the execution time (speed of execution) for different methods, including the proposed method, for different numbers of metabolites and samples (Supplementary Information Table S10). The URL of the R package and the user manual of our proposed method are https://github.com/NishithPaul/tWLSA.

## Conclusion

The Selection of the missing imputation method affects consecutive metabolomics data analysis. Moreover, metabolomics data generated from different platforms often contain missing values and outliers. Thus, in this study, we developed a new outlier-robust kernel-weight-based two-way alternating weighted least square approach for imputing missing values. We also measured the performance of our proposed KMI technique compared to the existing conventional methods (zero, mean, median, half of the minimum value, kNN, BPCA, PPCA, EM-PCA, and RF imputations) and recently developed missing imputation methods (GSimp, QRILC, BayesMetab, rmiMAE, and MICE) through both artificial and real metabolomics data analysis. Based on our computational results, the presently developed missing value imputation method is better than the existing missing value imputation methods in both the absence and presence of outliers. For this reason, our recommendation is to apply our proposed two-way kernel weighted least square-based missing value imputation method instead of existing missing imputation methods to substitute the missing values in metabolomics datasets for consecutive univariate, multivariate, and exploratory metabolomics data analysis.

## Supplementary information

Supplementary Informations.

## Data Availability

Comparisons were evaluated using R language. To identify the DE metabolites in R we used ‘*t.test*’ function (for two groups) and ‘*anova*’ function (for more than two groups) from “stats” package. “ROCR” and “pROC” packages have been used to draw receiver operating characteristic (ROC) curve as well as to calculate the area under the ROC curve. We also used the support vector machine ‘*svm*’ function from “e1071” package to calculate the misclassification error rate (MER) for sample classification. Moreover, The source code and packages of different missing imputation techniques are given below, **Proposed**: R package and the user manual of our proposed method are available at https://github.com/NishithPaul/tWLSA. **rmiMAE**: The R code for rmiMAE is available at https://github.com/NishithPaul/missingImputation/blob/main/rmiMAE.R. **GSimp**: The R code for GSimp is available at https://github.com/WandeRum/GSimp. **kNN:** Here, kNN imputation technique has been implemented using *"impute"* package from bioconductor. The reference manual of this package can be found at https://www.bioconductor.org/packages/release/bioc/manuals/impute/man/impute.pdf. **EM-PCA**: We used *"missMDA"* package in R to impute missing values by EM-PCA method. The reference manual of this package can be found at https://cran.r-project.org/web/packages/missMDA/missMDA.pdf. **RF:** *"missForest"* package has been used to impute the missing values by Random Forest (RF) method. The reference manual of this package can be found at https://cran.r-project.org/web/packages/missForest/missForest.pdf. **BPCA and PPCA:** BPCA and PPCA imputation techniques have been implemented using *"pcaMethods"* package from bioconductor. The reference manual is available at https://www.bioconductor.org/packages/release/bioc/manuals/pcaMethods/man/pcaMethods.pdf. **QRILC:** Here QRILC imputation technique has been implemented using “imputeLCMD” package in R. The reference manual can be found at https://cran.r-project.org/web/packages/imputeLCMD/imputeLCMD.pdf. **BayesMetab**: R code for BayesMetab method is available at https://bmcbioinformatics.biomedcentral.com/articles/10.1186/s12859-019-3250-2. **MICE:** Here, “missCompare” package in R has been used to impute the missing values by MICE method. The reference manual of the package can be found at https://cran.r-project.org/web/packages/missCompare/missCompare.pdf
