# Supplementary material for: Kernel weighted least square approach for imputing missing values of metabolomics data
Source: Sci Rep. 2021 May 27;11:11108. doi: 10.1038/s41598-021-90654-0 (PMC8159923; doi:10.1038/s41598-021-90654-0)
Supplement: Supplementary file 1 — Supplementary Informations. [file 41598_2021_90654_MOESM1_ESM.docx]

**Supplementary Information for**

**Kernel Weighted Least Square Approach for Missing Value Imputation of Metabolomics Data**

Nishith Kumar^1*^, Md. Aminul Hoque^2^, Masahiro Sugimoto^3^

^1^Department of Statistics, Bangabandhu Sheikh Mujibur Rahman Science and Technology University, Gopalganj, Bangladesh

^2^Department of Statistics, University of Rajshahi, Rajshahi, Bangladesh

^3^Health Promotion and Preemptive Medicine, Research and Development Center for Minimally Invasive Therapies, Tokyo Medical University, Shinjuku, Tokyo 160-8402, Japan

*** Corresponding author:** Nishith Kumar.

Department of Statistics, Bangabandhu Sheikh Mujibur Rahman Science and Technology University, Gopalganj, Bangladesh, E-mail: nk.bru09@gmail.com; Tel.: +88-01925200899

**Appendix-1**

**Appropriate *r* Selection using SVD**

Let the reconstructed data matrix of *X* be

$\hat{X}^{(r)}=$ $\sum_{k=1}^{r} m_{k}s_{k}\approx X$ (A1)

Using the singular value decomposition, we can write *X* as

$X=UDV^{T}$ (A2)

where *U* is the column orthogonal matrix that contains left singular vectors, *D* is a diagonal matrix that contains singular values, and *V* is the row orthogonal matrix that contains the right singular vectors. From equation -A2,

$X=\sum_{i=1}^{r} d_{i}u_{i}v_{i}$ ; $r\leq\min(n,p)$ (A3)

Again from equation -A2, we can write$X^{T}X=VDU^{T}UDV^{T}=VD^{2}V^{T}$. Therefore, the right singular vector is the characteristic vector of$X^{T}X$, and the square of the singular value is the characteristic root of$X^{T}X$.

Now, using equations (A1) and (A3),

$$X=\sum_{i=1}^{r} d_{i}u_{i}v_{i}\approx$$

$$\sum_{k=1}^{r} m_{k}s_{k}$$

$$=\sum_{k=1}^{r} \left(||m_{k}||.||s_{k}||\right)\left(\frac{m_{k}}{||m_{k}||}\right)\left(\frac{s_{k}}{||s_{k}||}\right)$$

$=\sum_{k=1}^{r} \hat{d}_{\text{k}}\hat{\text{u}}_{k}\hat{\text{v}}_{k}$ ; where $\hat{d}_{\text{k}}$=$\left(||m_{k}||.||s_{k}||\right)$; $\hat{\text{u}}_{k}$=$\left(\frac{m_{k}}{||m_{k}||}\right)$; $\hat{\text{v}}_{k}$=$\left(\frac{s_{k}}{||s_{k}||}\right)$

Choose r such that the first r singular values can explain at least (1-*α*)100*%* variation of row (metabolites) effect, that is,$\frac{\sum_{k=1}^{r} (d_{k}^{*})^{2}}{\sum_{i=1}^{p} V(x_{i})}\geq\left(1-\alpha\right)100\%$, where *V*(*x_i_*) is the variance of the *i*-th row (metabolite) and $i=1,2,\cdots,p.$


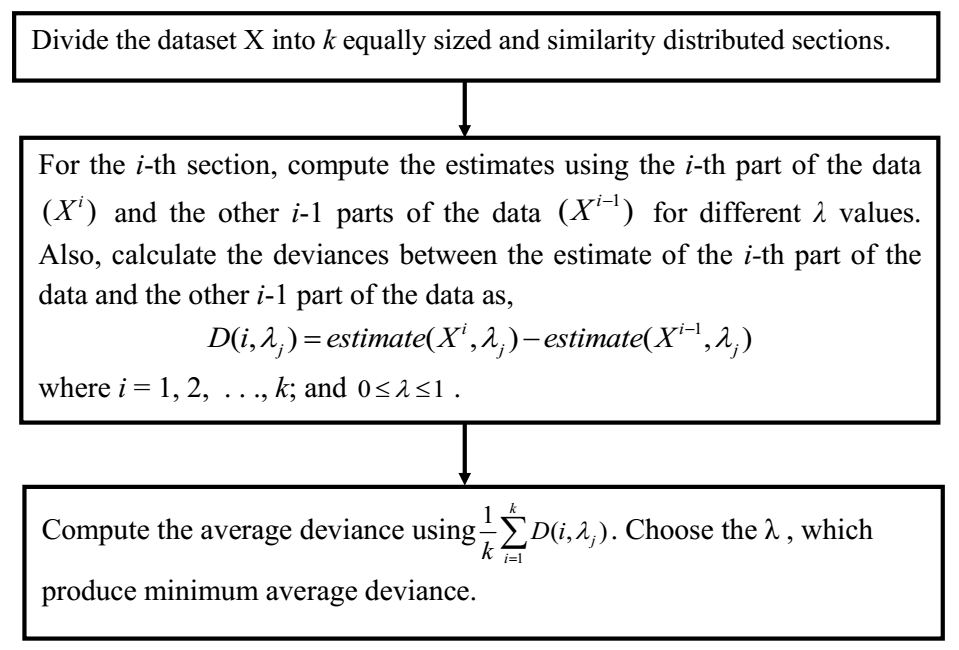


**Diagram S1.** *λ* selection procedure

**Table S1.** Average misclassification error rate (MER) and area under the receiver operating characteristic curve (AUC) of DE calculation for three class simulated data with 5% missing values and different rates of outliers.

| **Methods** | **Without Outliers**  **MER (AUC)** | **3% Outliers**  **MER (AUC)** | **5% Outliers**  **MER (AUC)** | **7% Outliers**  **MER (AUC)** | **10% Outliers**  **MER (AUC)** |
| --- | --- | --- | --- | --- | --- |
| **RF** | 1.42 (0.986) | 12.61 (0.871) | 19.49 (0.802) | 23.12 (0.766) | 27.64 (0.719) |
| **PPCA** | 1.29 (0.987) | 11.54 (0.885) | 18.02 (0.820) | 21.76 (0.782) | 26.33 (0.734) |
| **kNN** | 1.91 (0.981) | 13.31 (0.867) | 20.26 (0.794) | 23.76 (0.759) | 28.24 (0.714) |
| **BPCA** | 1.76 (0.983) | 13.30 (0.872) | 20.21 (0.806) | 23.76 (0.771) | 28.23 (0.718) |
| **EM-PCA** | 1.26 (0.988) | 11.61 (0.884) | 18.08 (0.826) | 21.78 (0.801) | 26.27 (0.737) |
| **Zero** | 10.89 (0.885) | 21.79 (0.775) | 27.64 (0.722) | 30.74 (0.687) | 33.91 (0.662) |
| **Mean** | 2.23 (0.978) | 14.32 (0.856) | 21.03 (0.791) | 24.57 (0.756) | 28.89 (0.713) |
| **Median** | 2.09 (0.979) | 13.93 (0.857) | 20.78 (0.792) | 24.15 (0.752) | 28.73 (0.723) |
| **Minimum** | 7.14 (0.924) | 19.02 (0.808) | 25.27 (0.739) | 28.69 (0.712) | 32.33 (0.673) |
| **rmiMAE** | 1.43 (0.986) | 1.97 (0.981) | 2.52 (0.974) | 2.91 (0.969) | 4.68 (0.954) |
| **Proposed** | **1.18 (0.988)** | **1.48 (0.985)** | **1.57 (0.984)** | **1.81 (0.982)** | **3.15 (0.969)** |

**Table S2.** Average misclassification error rate (MER) and area under the receiver operating characteristic curve (AUC) for two class simulated data with 10% missing values and different rates of outliers.

| **Methods** | **Without Outliers**  **MER (AUC)** | **3% Outliers**  **MER (AUC)** | **5% Outliers**  **MER (AUC)** | **7% Outliers**  **MER (AUC)** | **10% Outliers**  **MER (AUC)** |
| --- | --- | --- | --- | --- | --- |
| **RF** | 4.39 (0.955) | 23.08 (0.767) | 28.22 (0.714) | 31.91 (0.675) | 36.01 (0.633) |
| **PPCA** | 3.89 (0.959) | 20.95 (0.790) | 25.76 (0.739) | 29.62 (0.701) | 33.68 (0.660) |
| **kNN** | 5.27 (0.946) | 24.29 (0.754) | 29.16 (0.704) | 32.68 (0.679) | 36.47 (0.639) |
| **BPCA** | 3.72 (0.965) | 26.03 (0.747) | 30.96 (0.699) | 34.03 (0.667) | 37.48 (0.631) |
| **EM-PCA** | 4.06 (0.961) | 20.46 (0.802) | 25.13 (0.743) | 28.94 (0.716) | 33.24 (0.665) |
| **Zero** | 27.82 (0.718) | 37.71 (0.605) | 40.06 (0.594) | 41.62 (0.584) | 43.50 (0.574) |
| **Mean** | 6.35 (0.938) | 26.01 (0.742) | 30.99 (0.694) | 33.95 (0.665) | 37.44 (0.631) |
| **Median** | 6.13 (0.939) | 25.79 (0.748) | 30.67 (0.691) | 33.88 (0.663) | 37.31 (0.632) |
| **Minimum** | 21.07 (0.783) | 34.46 (0.658) | 37.53 (0.624) | 39.46 (0.605) | 41.92 (0.582) |
| **rmiMAE** | 3.11 (0.969) | 4.37 (0.957) | 4.89 (0.952) | 5.18 (0.949) | 5.62 (0.943) |
| **Proposed** | **2.98 (0.971)** | **3.01 (0.971)** | **3.09 (0.970)** | **3.16 (0.969)** | **3.49 (0.967)** |

**Table S3.** Average misclassification error rate (MER) and area under the receiver operating characteristic curve (AUC) for two class simulated data with 15% missing values and different rates of outliers.

| **Methods** | **Without Outliers**  **MER (AUC)** | **3% Outliers**  **MER (AUC)** | **5% Outliers**  **MER (AUC)** | **7% Outliers**  **MER (AUC)** | **10% Outliers**  **MER (AUC)** |
| --- | --- | --- | --- | --- | --- |
| **RF** | 4.42 (0.954) | 24.38 (0.751) | 29.53 (0.699) | 33.31 (0.663) | 37.22 (0.621) |
| **PPCA** | 3.95 (0.967) | 21.42 (0.785) | 25.97 (0.737) | 29.96 (0.698) | 34.08 (0.658) |
| **kNN** | 5.460 (0.944) | 25.98 (0.741) | 30.59 (0.691) | 34.35 (0.653) | 37.99 (0.616) |
| **BPCA** | 3.75 (0.968) | 28.48 (0.723) | 32.93 (0.677) | 36.07 (0.639) | 39.16 (0.608) |
| **EM-PCA** | 4.19 (0.965) | 21.85 (0.791) | 26.22 (0.742) | 30.01 (0.707) | 33.97 (0.661) |
| **Zero** | 33.35 (0.663) | 40.82 (0.592) | 42.62 (0.572) | 44.27 (0.558) | 45.41 (0.543) |
| **Mean** | 7.80 (0.926) | 28.41 (0.709) | 33.03 (0.669) | 36.22 (0.644) | 39.20 (0.606) |
| **Median** | 7.16 (0.928) | 28.16 (0.715) | 32.59 (0.676) | 35.97 (0.641) | 38.97 (0.619) |
| **Minimum** | 26.17 (0.729) | 37.45 (0.616) | 40.16 (0.596) | 42.35 (0.574) | 43.84 (0.559) |
| **rmiMAE** | 3.54 (0.964) | 4.49 (0.955) | 5.78 (0.943) | 5.99 (0.941) | 6.41 (0.935) |
| **Proposed** | **3.05 (0.969)** | **3.05 (0.969)** | **3.46 (0.965)** | **3.78 (0.963)** | **4.01 (0.959)** |

**Table S4.** Average misclassification error rate (MER) and area under the receiver operating characteristic curve (AUC) for two class simulated data with 20% missing values and different rates of outliers.

| **Methods** | **Without Outliers**  **MER (AUC)** | **3% Outliers**  **MER (AUC)** | **5% Outliers**  **MER (AUC)** | **7% Outliers**  **MER (AUC)** | **10% Outliers**  **MER (AUC)** |
| --- | --- | --- | --- | --- | --- |
| **RF** | 4.68 (0.952) | 25.78 (0.737) | 31.39 (0.682) | 34.99 (0.649) | 38.68 (0.607) |
| **PPCA** | 3. 61 (0.965) | 21.46 (0.785) | 26.37 (0.736) | 30.54 (0.692) | 34.69 (0.648) |
| **kNN** | 6.28 (0.936) | 27.53 (0.720) | 32.29 (0.673) | 35.71 (0.637) | 39.17 (0.601) |
| **BPCA** | 9.96 (0.903) | 30.92 (0.699) | 34.99 (0.658) | 37.89 (0.629) | 40.80 (0.593) |
| **EM-PCA** | 4.44 (0.959) | 22.49 (0.776) | 26.71 (0.736) | 30.79 (0.701) | 34.52 (0.658) |
| **Zero** | 38.43 (0.614) | 43.48 (0.574) | 44.84 (0.548) | 45.84 (0.548) | 46.78 (0.535) |
| **Mean** | 9.90 (0.894) | 30.86 (0.699) | 35.03 (0.656) | 38.03 (0.618) | 40.93 (0.596) |
| **Median** | 9.00 (0.901) | 30.38 (0.693) | 34.70 (0.659) | 37.65 (0.621) | 40.73 (0.594) |
| **Minimum** | 31.46 (0.681) | 40.52 (0.593) | 42.87 (0.579) | 44.17 (0.556) | 45.69 (0.538) |
| **rmiMAE** | 3.95 (0.961) | 5.19 (0.948) | 6.28 (0.938) | 6.49 (0.937) | 7.11 (0.929) |
| **Proposed** | **3.60 (0.965)** | **3.64 (0.965)** | **3.75 (0.962)** | **3.54 (0.964)** | **5.25 (0.948)** |

**Table S5.** Average misclassification error rate (MER) and area under the receiver operating characteristic curve (AUC) for three class simulated data with 10% missing values and different rates of outliers.

| **Methods** | **Without Outliers**  **MER (AUC)** | **3% Outliers**  **MER (AUC)** | **5% Outliers**  **MER (AUC)** | **7% Outliers**  **MER (AUC)** | **10% Outliers**  **MER (AUC)** |
| --- | --- | --- | --- | --- | --- |
| **RF** | 1.74 (0.982) | 14.16 (0.856) | 21.20 (0.785) | 25.37 (0.742) | 29.62 (0.699) |
| **PPCA** | 1.31 (0.987) | 11.87 (0.882) | 18.69 (0.811) | 22.21 (0.776) | 26.88 (0.728) |
| **kNN** | 2.74 (0.971) | 15.49 (0.844) | 22.61 (0.773) | 26.32 (0.735) | 30.67 (0.691) |
| **BPCA** | 2.58 (0.975) | 15.58 (0.850) | 22.67 (0.781) | 26.13 (0.747) | 30.61 (0.702) |
| **EM-PCA** | 1.39 (0.988) | 12.05 (0.889) | 18.63 (0.829) | 22.09 (0.787) | 26.75 (0.736) |
| **Zero** | 23.30 (0.768) | 31.90 (0.679) | 35.81 (0.639) | 37.79 (0.623) | 40.46 (0.599) |
| **Mean** | 3.89 (0.961) | 17.51 (0.828) | 24.51 (0.759) | 28.00 (0.724) | 31.94 (0.685) |
| **Median** | 3.51 (0.963) | 17.01 (0.831) | 24.02 (0.758) | 27.71 (0.723) | 31.75 (0.681) |
| **Minimum** | 16.47 (0.833) | 27.45 (0.724) | 32.45 (0.672) | 35.06 (0.643) | 38.11 (0.617) |
| **rmiMAE** | 1.61 (0.984) | 2.99 (0.969) | 3.24 (0.966) | 4.12 (0.958) | 6.15 (0.938) |
| **Proposed** | **1.20 (0.988)** | **2.05 (0.979)** | **2.07 (0.979)** | **2.58 (0.974)** | **4.81 (0.955)** |

**Table S6.**Average misclassification error rate (MER) and area under the receiver operating characteristic curve (AUC) for three class simulated data with 15% missing values and different rates of outliers.

| **Methods** | **Without Outliers**  **MER (AUC)** | **3% Outliers**  **MER (AUC)** | **5% Outliers**  **MER (AUC)** | **7% Outliers**  **MER (AUC)** | **10% Outliers**  **MER (AUC)** |
| --- | --- | --- | --- | --- | --- |
| **RF** | 2.08 (0.978) | 15.66 (0.842) | 23.81 (0.758) | 27.44 (0.721) | 31.77 (0.678) |
| **PPCA** | 1.39 (0.986) | 12.45 (0.874) | 19.72 (0.802) | 23.31 (0.764) | 27.96 (0.717) |
| **kNN** | 3.83 (0.961) | 17.61 (0.824) | 25.25 (0.747) | 28.65 (0.713) | 32.61 (0.674) |
| **BPCA** | 3.66 (0.964) | 17.80 (0.827) | 25.16 (0.755) | 28.79 (0.721) | 32.75 (0.682) |
| **EM-PCA** | 1.77 (0.984) | 12.79 (0.875) | 19.77 (0.804) | 23.31 (0.765) | 27.77 (0.726) |
| **Zero** | 29.88 (0.706) | 36.64 (0.631) | 40.00 (0.599) | 41.59 (0.582) | 43.06 (0.577) |
| **Mean** | 6.28 (0.936) | 20.78 (0.794) | 27.75 (0.726) | 30.91 (0.695) | 34.51 (0.660) |
| **Median** | 5.41 (0.948) | 20.06 (0.797) | 27.17 (0.727) | 30.31 (0.695) | 34.29 (0.657) |
| **Minimum** | 22.28 (0.773) | 32.05 (0.679) | 36.55 (0.637) | 38.58 (0.615) | 41.06 (0.584) |
| **rmiMAE** | 1.96 (0.981) | 3.93 (0.961) | 4.46 (0.955) | 5.51 (0.946) | 8.54 (0.916) |
| **Proposed** | **1.24 (0.988)** | **2.91 (0.971)** | **3.02 (0.971)** | **3.77 (0.963)** | **6.83 (0.934)** |

**Table S7.**Average misclassification error rate (MER) and area under the receiver operating characteristic curve (AUC) for three class simulated data with 20% missing values and different rates of outliers.

| **Methods** | **Without Outliers**  **MER (AUC)** | **3% Outliers**  **MER (AUC)** | **5% Outliers**  **MER (AUC)** | **7% Outliers**  **MER (AUC)** | **10% Outliers**  **MER (AUC)** |
| --- | --- | --- | --- | --- | --- |
| **RF** | 2.68 (0.973) | 18.39 (0.813) | 26.09 (0.734) | 29.79 (0.697) | 33.65 (0.657) |
| **PPCA** | 1.98 (0.981) | 14.03 (0.858) | 20.81 (0.789) | 24.56 (0.751) | 28.95 (0.708) |
| **kNN** | 5.48 (0.945) | 20.76 (0.791) | 27.59 (0.722) | 30.96 (0.689) | 34.69 (0.652) |
| **BPCA** | 5.37 (0.947) | 21.07 (0.796) | 27.62 (0.733) | 30.94 (0.691) | 34.62 (0.654) |
| **EM-PCA** | 2.35 (0.979) | 14.38 (0.859) | 20.89 (0.789) | 24.46 (0.751) | 28.69 (0.717) |
| **Zero** | 35.45 (0.644) | 40.72 (0.593) | 42.84 (0.571) | 43.96 (0.558) | 45.21 (0.541) |
| **Mean** | 9.71 (0.904) | 24.92 (0.753) | 31.00 (0.694) | 33.77 (0.667) | 36.91 (0.636) |
| **Median** | 8.48 (0.912) | 24.24 (0.754) | 30.47 (0.691) | 33.41 (0.662) | 36.55 (0.633) |
| **Minimum** | 28.63 (0.712) | 36.96 (0.634) | 40.09 (0.598) | 41.85 (0.582) | 43.66 (0.562) |
| **rmiMAE** | 2.28 (0.973) | 5.42 (0.946) | 6.21 (0.937) | 7.34 (0.925) | 11.06 (0.890) |
| **Proposed** | **1.52 (0.984)** | **4.24 (0.958)** | **4.60 (0.955)** | **5.88 (0.943)** | **9.40 (0.911)** |

**Table S8.** Average misclassification error rate (MER) and area under the receiver operating characteristic curve (AUC) for two class simulated data with 10% missing values and different rates of outliers.

| **Methods** | **3% Outliers**  **MER (AUC)** | **5% Outliers**  **MER (AUC)** | **7% Outliers**  **MER (AUC)** | **10% Outliers**  **MER (AUC)** |
| --- | --- | --- | --- | --- |
| **RF** | 4.7 (0.9536) | 13.03 (0.8691) | 14.20 (0.8576) | 22.20 (0.7672) |
| **PPCA** | 7.93 (0.9204) | 11.77 (0.8831) | 13.20 (0.8672) | 21.90 (0.7799) |
| **kNN** | 8.73 (0.9108) | 15.67 (0.8412) | 16.43 (0.8360) | 19.93 (0.7915) |
| **BPCA** | 5.97 (0.9394) | 15.43 (0.8449) | 13.43 (0.8654) | 22.43 (0.7599) |
| **EM-PCA** | 4.67 (0.9529) | 11.53 (0.8821) | 13.23 (0.8661) | 22.17 (0.7624) |
| **Zero** | 12.40 (0.8723) | 17.33 (0.8246) | 23.70 (0.7636) | 18.33 (0.8018) |
| **Mean** | 11.03 (0.8873) | 15.20 (0.8467) | 13.77 (0.8623) | 20.07 (0.7853) |
| **Median** | 11.03 (0.8876) | 14.56 (0.8571) | 14.17 (0.8584) | 20.13 (0.7814) |
| **Minimum** | 13.97 (0.8541) | 14.73 (0.8569) | 15.87 (0.8409) | 18.50 (0.7988) |
| **rmiMAE** | 2.17 (0.9782) | 2.38 (0.9763) | 2.51 (0.9758) | 2.86 ( 0.9712) |
| **Proposed** | **1.34 (0.9892)** | **1.39 (0.9873)** | **1.47 (0.9864)** | **1.56 (0.9835)** |

**Table S9.** average misclassification error rate (MER) and area under the receiver operating characteristic curve (AUC) for three class simulated data with 10% missing values and different rates of outliers.

| **Methods** | **3% Outliers**  **MER (AUC)** | **5% Outliers**  **MER (AUC)** | **7% Outliers**  **MER (AUC)** | **10% Outliers**  **MER (AUC)** |
| --- | --- | --- | --- | --- |
| **RF** | 6.57 (0.9539) | 11.57 (0.8729) | 17.50 (0.8324) | 25.40 (0.7324) |
| **PPCA** | 6.27 (0.9549) | 9.93 (0.9026) | 16.71 (0.8435) | 24.82 (0.7489) |
| **kNN** | 4.33 (0.9748) | 13.33 (0.8537) | 19.70 (0.8167) | 21.02 (0.7827) |
| **BPCA** | 4.45 (0.9729) | 10.80 (0.8845) | 15.80 (0.8497) | 24.82 (0.7488) |
| **EM-PCA** | 6.17 (0.9566) | 9.43 (0.9056) | 16.40 (0.8443) | 25.96 (0.7293) |
| **Zero** | 6.07 (0.9579) | 6.93 (0.9301) | 18.43 (0.8258) | 21.84 (0.7802) |
| **Mean** | 4.17 (0.9757) | 11.63 (0.8717) | 19.37 (0.8072) | 22.95 (0.7673) |
| **Median** | 4.23 (0.9754) | 11.40 (0.8735) | 18.60 (0.8201) | 20.97 (0.7920) |
| **Minimum** | 5.80 (0.9602) | 8.77 (0.9108) | 22.07 (0.7704) | 17.51 (0.8295) |
| **rmiMAE** | 2.31 (0.9768) | 2.46 (0.9753) | 3.15 (0.9685) | 3. 98 (0.9601) |
| **Proposed** | **1.37 (0.9872)** | **1.52 (0.9856)** | **1.74 (0.9837)** | **1.89 (0.9829)** |

**Table S10.** Execution time calculation in minute of different methods including the proposed one for different number of metabolites and different number of samples.

| Methods | No. of Metabolites=200  No. of Sample=90 | No. of Metabolites=200  No. of Sample=70 | No. No. of Metabolites=500  No. of Sample=90 | No. of Metabolites=500  No. of Sample=70 |
| --- | --- | --- | --- | --- |
| RF | 0.0349 | 0.0126 | 0.0685 | 0.0504 |
| PPCA | 0.004 | 0.0026 | 0.0151 | 0.0047 |
| KNN | 0.0014 | 0.0012 | 0.0016 | 0.0014 |
| BPCA | 0.0126 | 0.0123 | 0.0289 | 0.0231 |
| EMPCA | 0.0076 | 0.0033 | 0.0162 | 0.0091 |
| Zero | 0.0006 | 0.0005 | 0.0007 | 0.0006 |
| Mean | 0.0019 | 0.0018 | 0.0026 | 0.0023 |
| Median | 0.0016 | 0.0014 | 0.0019 | 0.0019 |
| Half minimum | 0.0014 | 0.0014 | 0.0019 | 0.0019 |
| Gsimp | 4.1494 | 2.8001 | 5.9121 | 3.9014 |
| BayesMetab | 9.2742 | 6.5283 | 13.4633 | 11.1127 |
| MICE | 20.6733 | 18.7471 | 27.3202 | 22.8614 |
| QRILC | 0.0035 | 0.0032 | 0.0068 | 0.0042 |
| rmiMAE | 0.1046 | 0.0708 | 0.2416 | 0.1654 |
| Proposed | 0.5811 | 0.4013 | 1.7832 | 1.0392 |

Computer Configuration: Processor-Intel Core i5 2.6 GHz, RAM-4.0GB, OS- 32 bit & Windows 7.


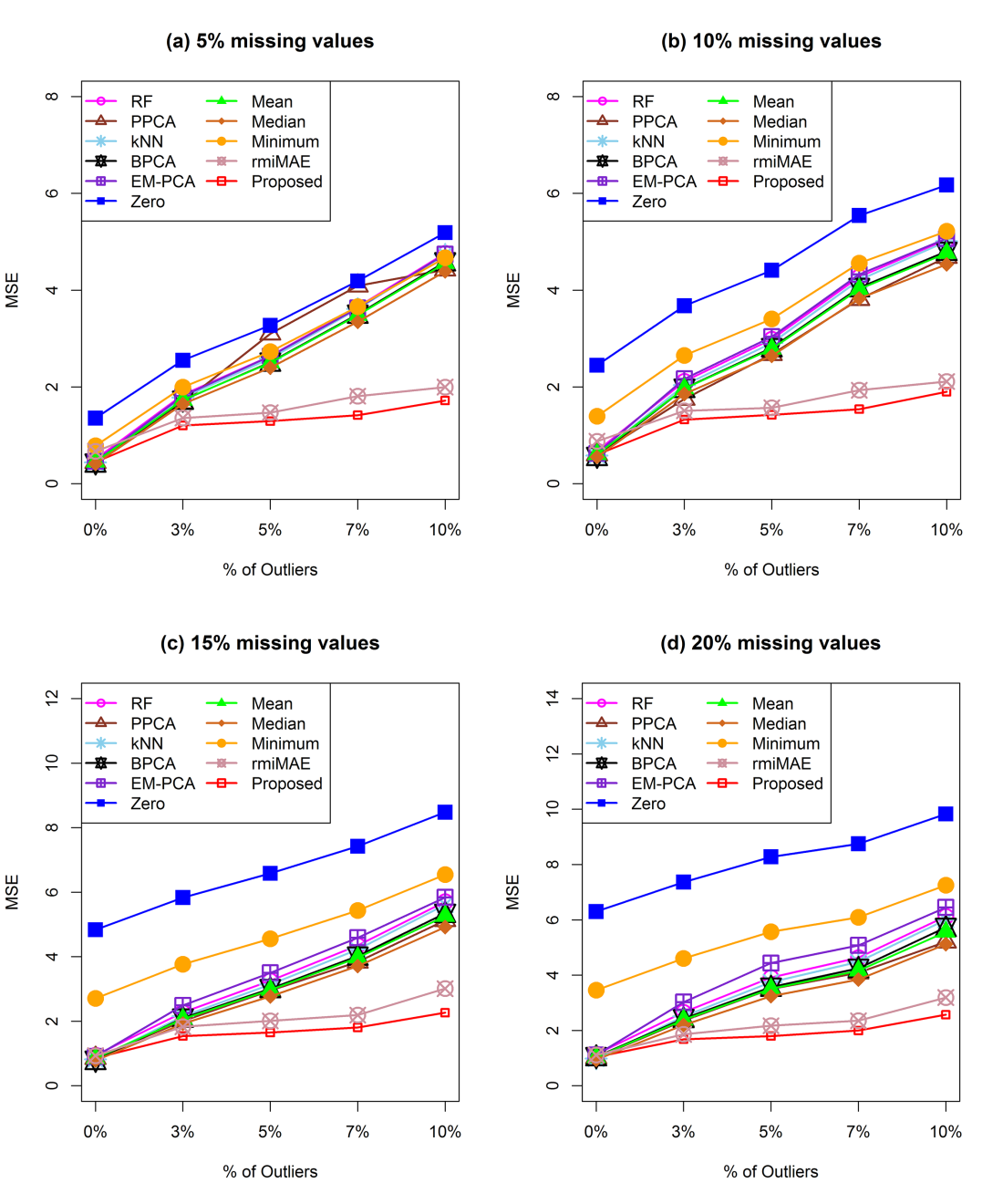


**Figure S1.** Performance investigation of different missing imputation techniques using average MSE for two class level data.


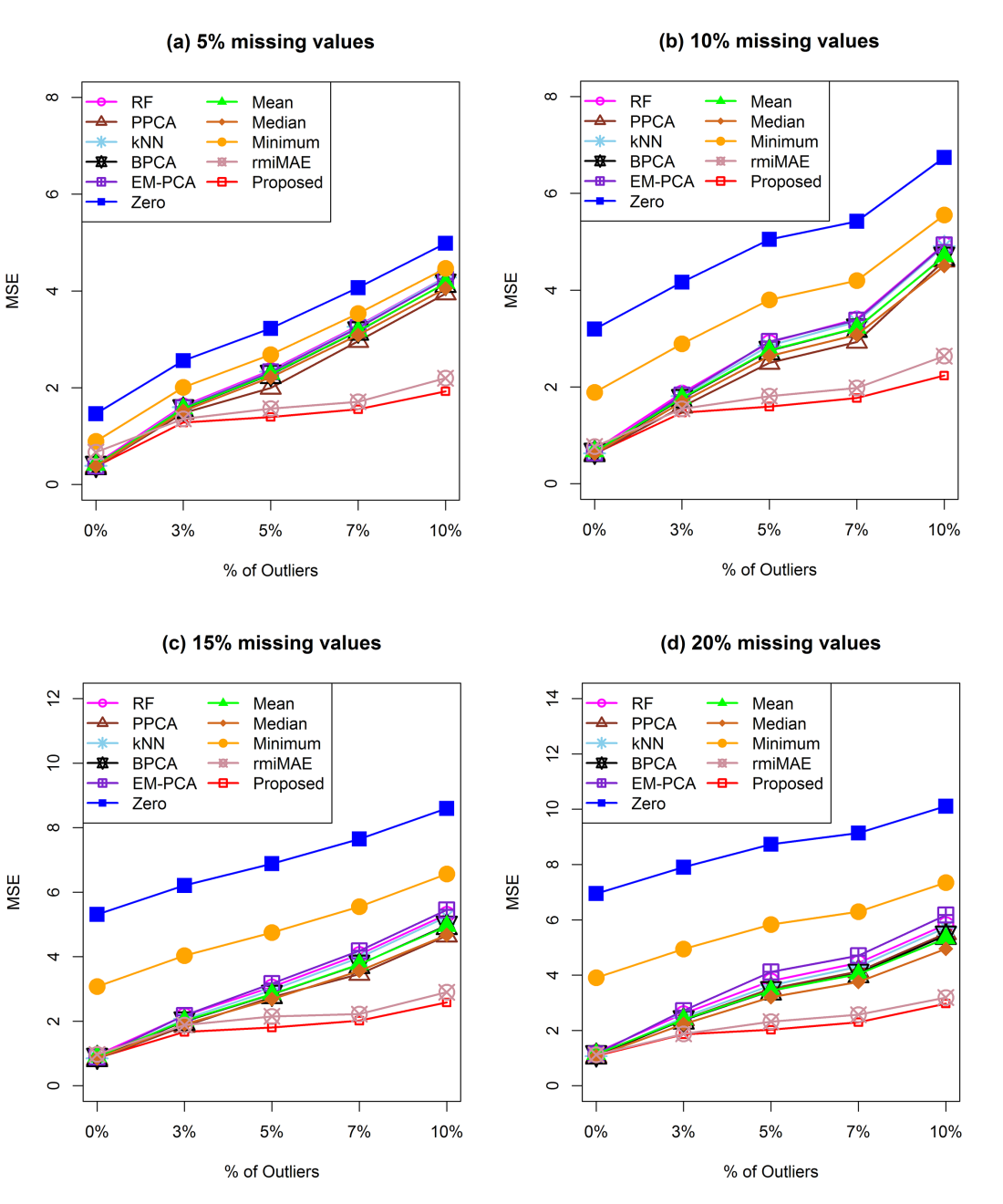


**Figure S2.** Performance investigation of different missing imputation techniques using average MSE for three class level data.


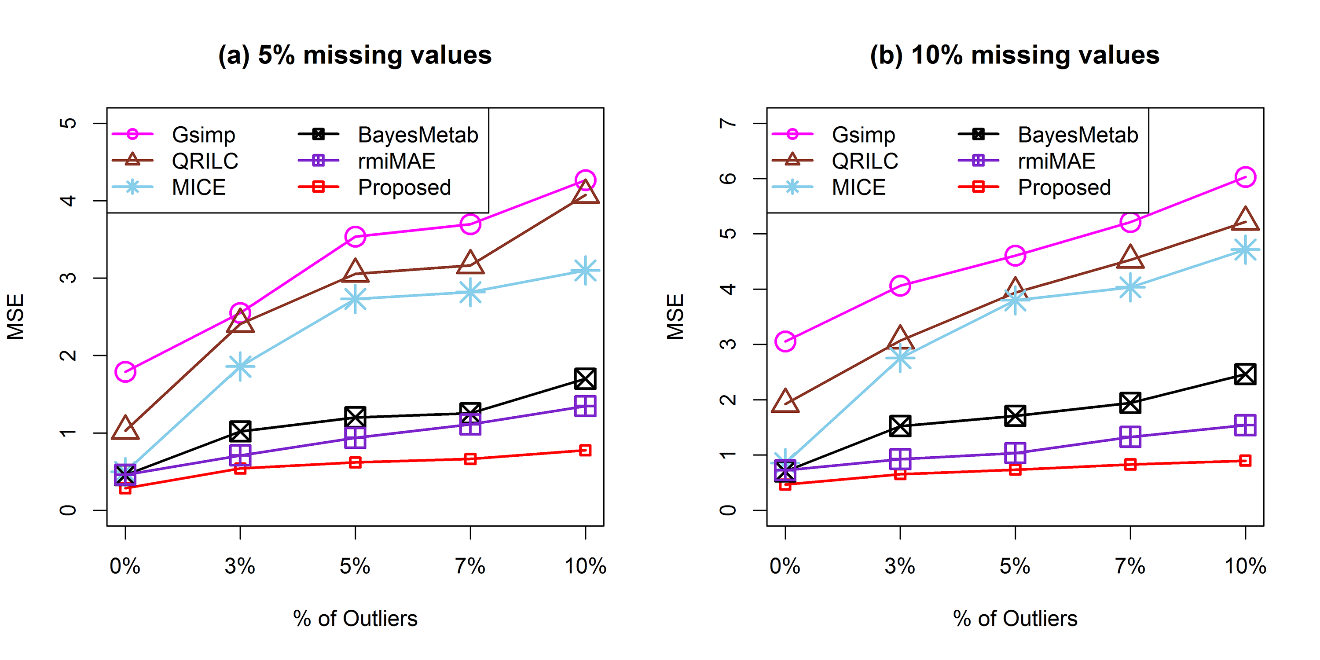


**Figure S3.** Performance investigation of different missing imputation techniques using average MSE.

**
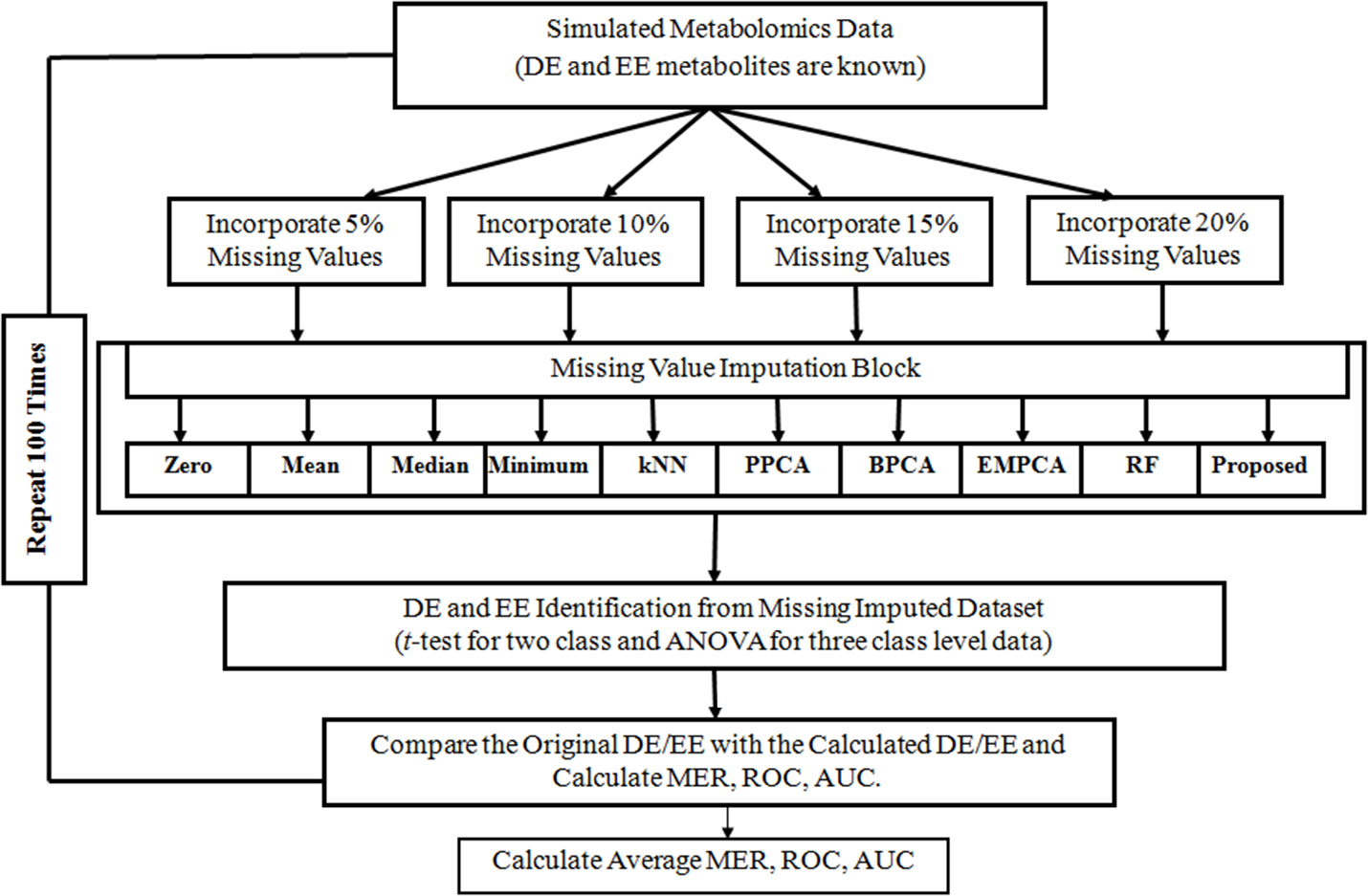
**

**Figure S4.**  Performance measures calculation procedure on the basis of DE calculation.


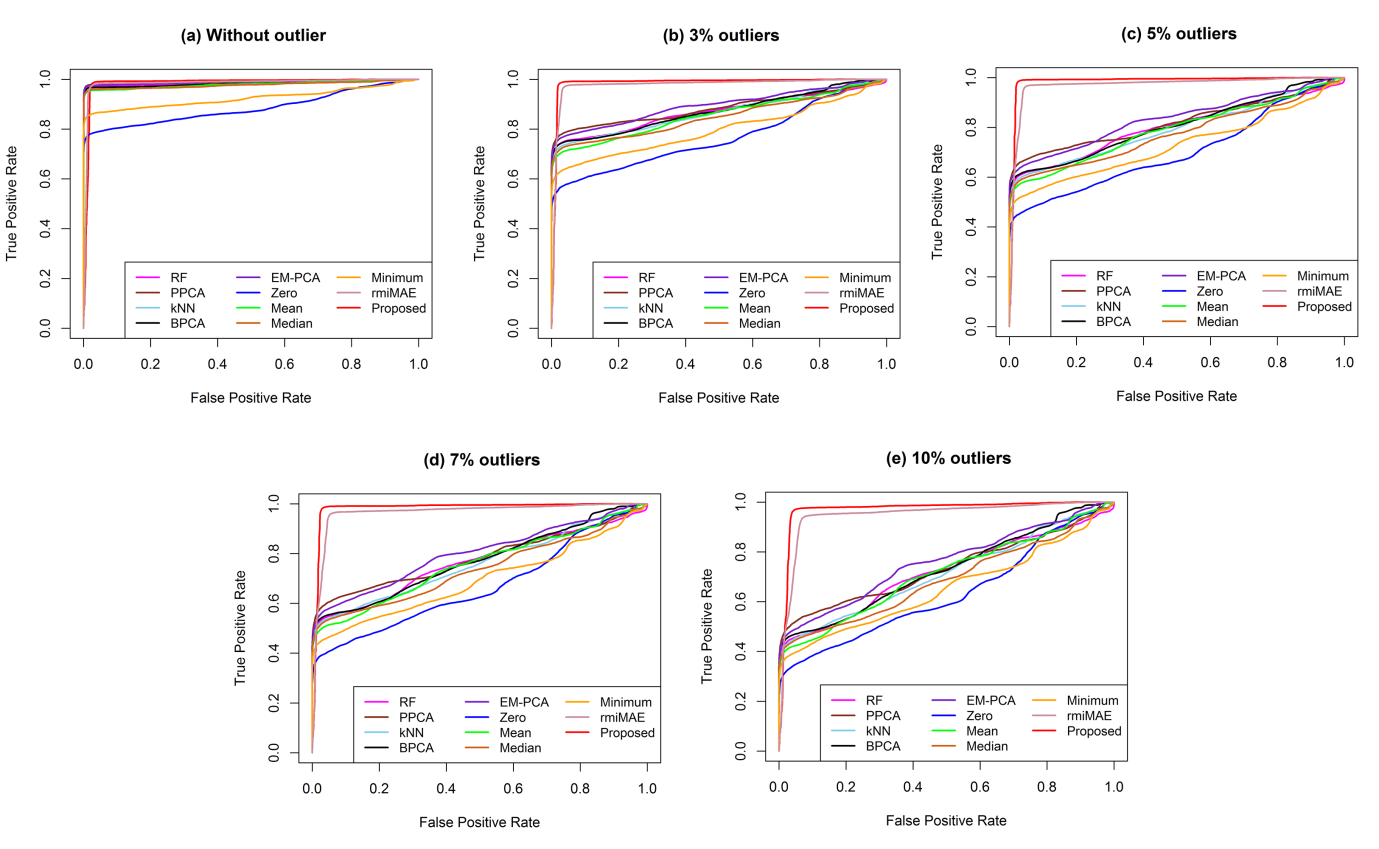


**Figure S5.** Performance investigation of different missing value imputation techniques using receiver operating characteristic curve of DE calculation for three class level dataset with 5% missing values in absence and presence of outliers.


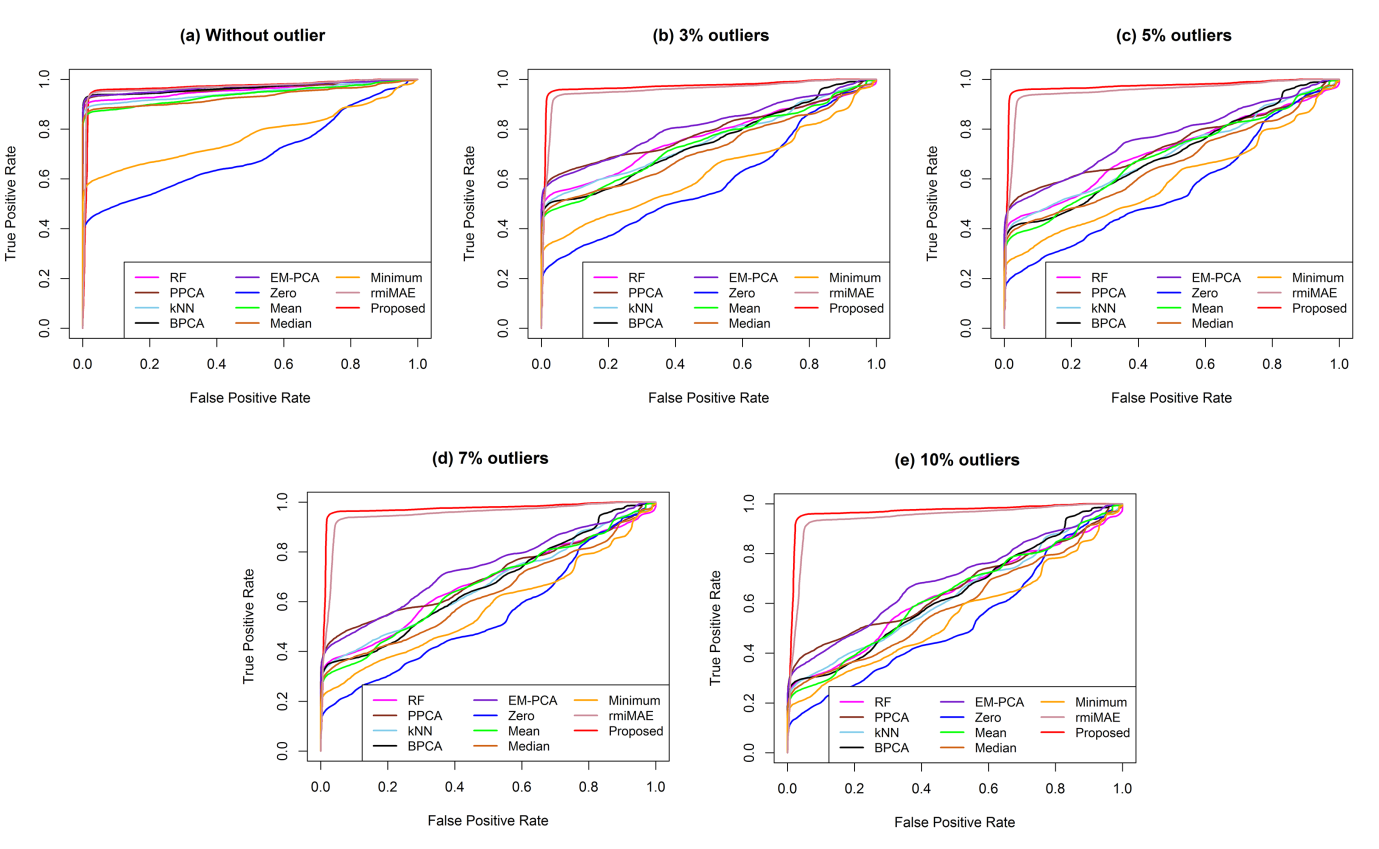


**Figure S6.**Performance investigation of different missing value imputation techniques using receiver operating characteristic curve of DE calculation for two class level dataset with 10% missing values in absence and presence of outliers.

**
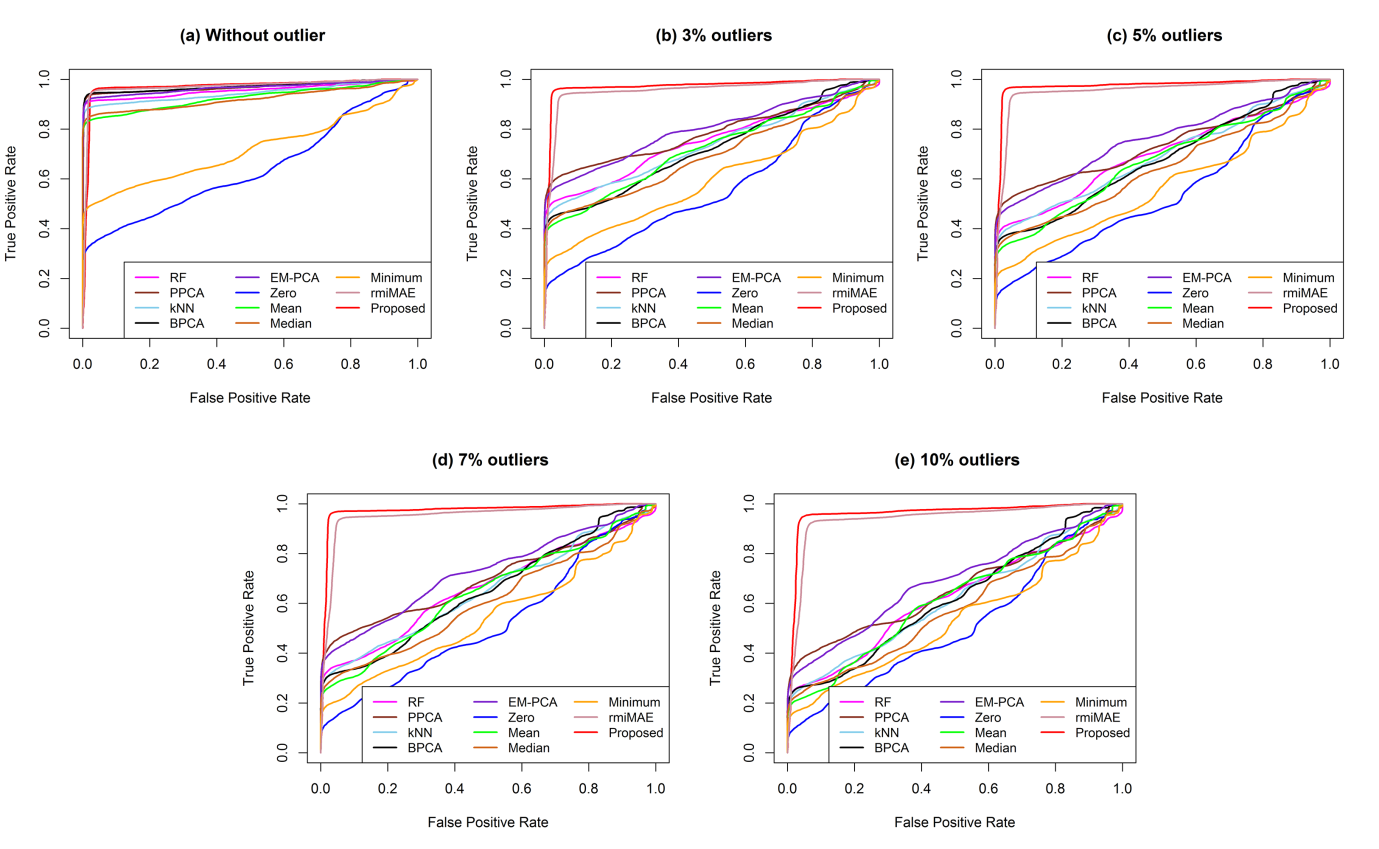
**

**Figure S7.** Performance investigation of different missing value imputation techniques using receiver operating characteristic curve of DE calculation for two class level dataset with 15% missing values in absence and presence of outliers.

**
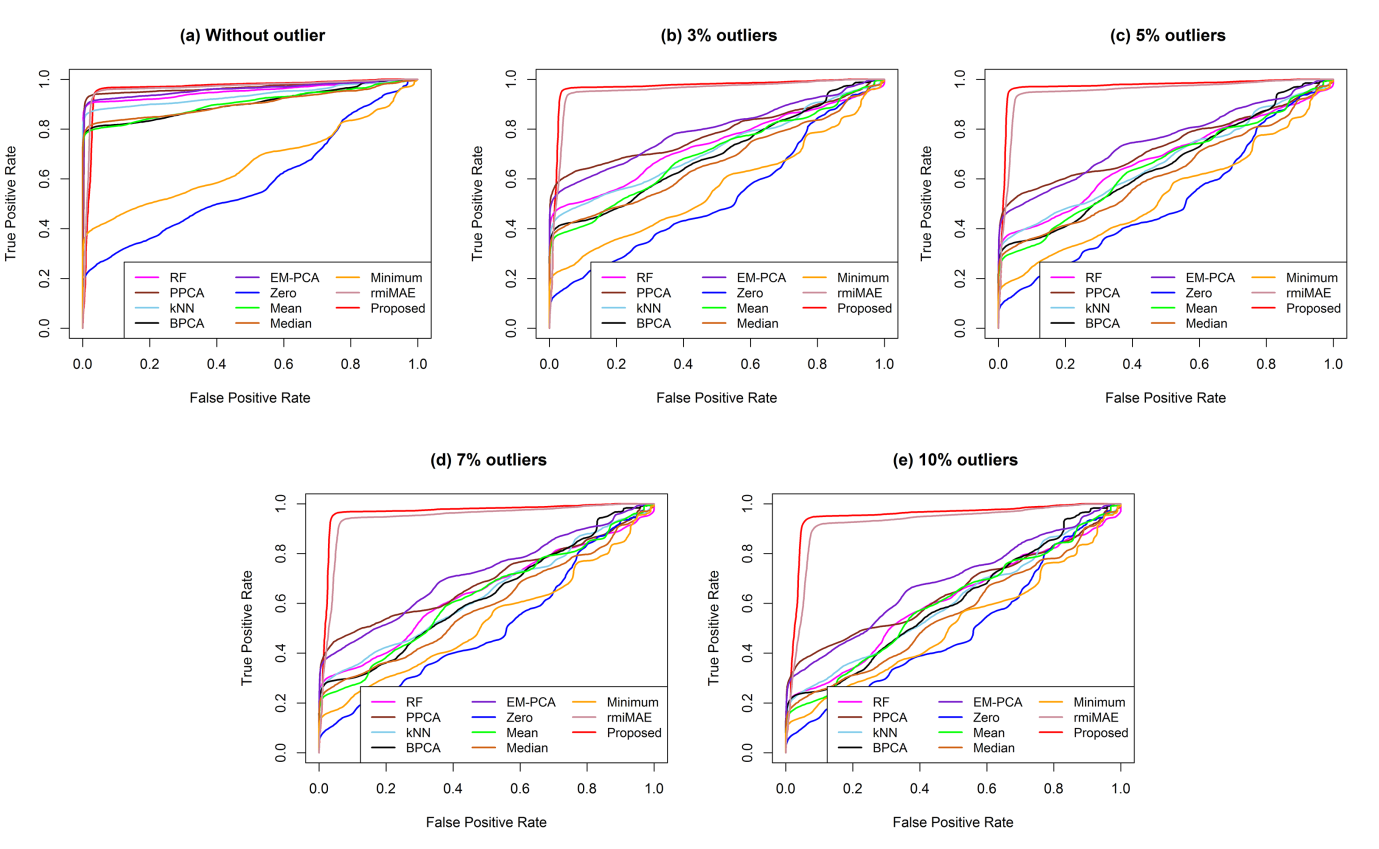
**

**Figure S8.**Performance investigation of different missing value imputation techniques using receiver operating characteristic curve of DE calculation for two class level dataset with 20% missing values in absence and presence of outliers.


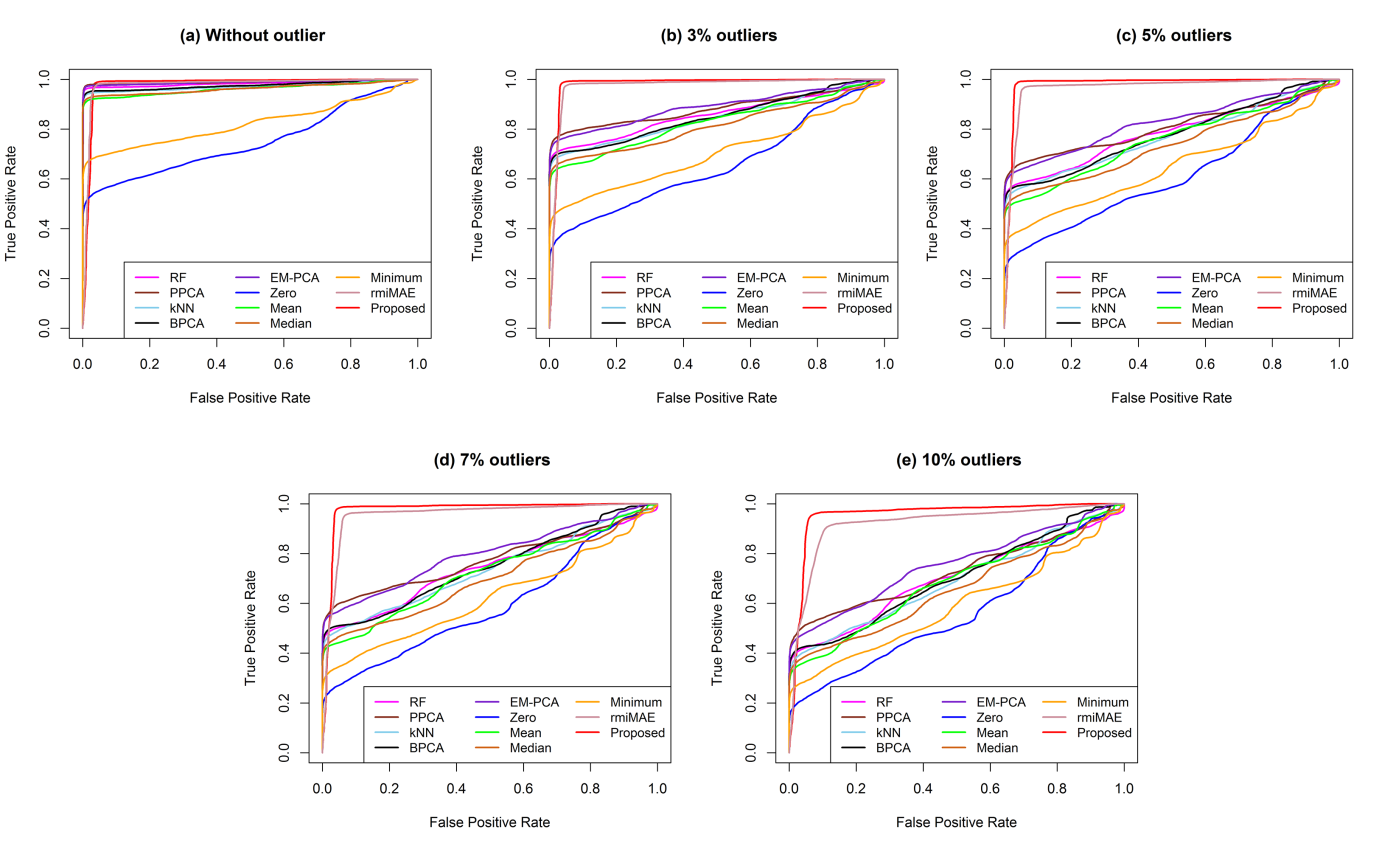


**Figure S9.**Performance investigation of different missing value imputation techniques using receiver operating characteristic curve of DE calculation for three class level dataset with 10% missing values in absence and presence of outliers.


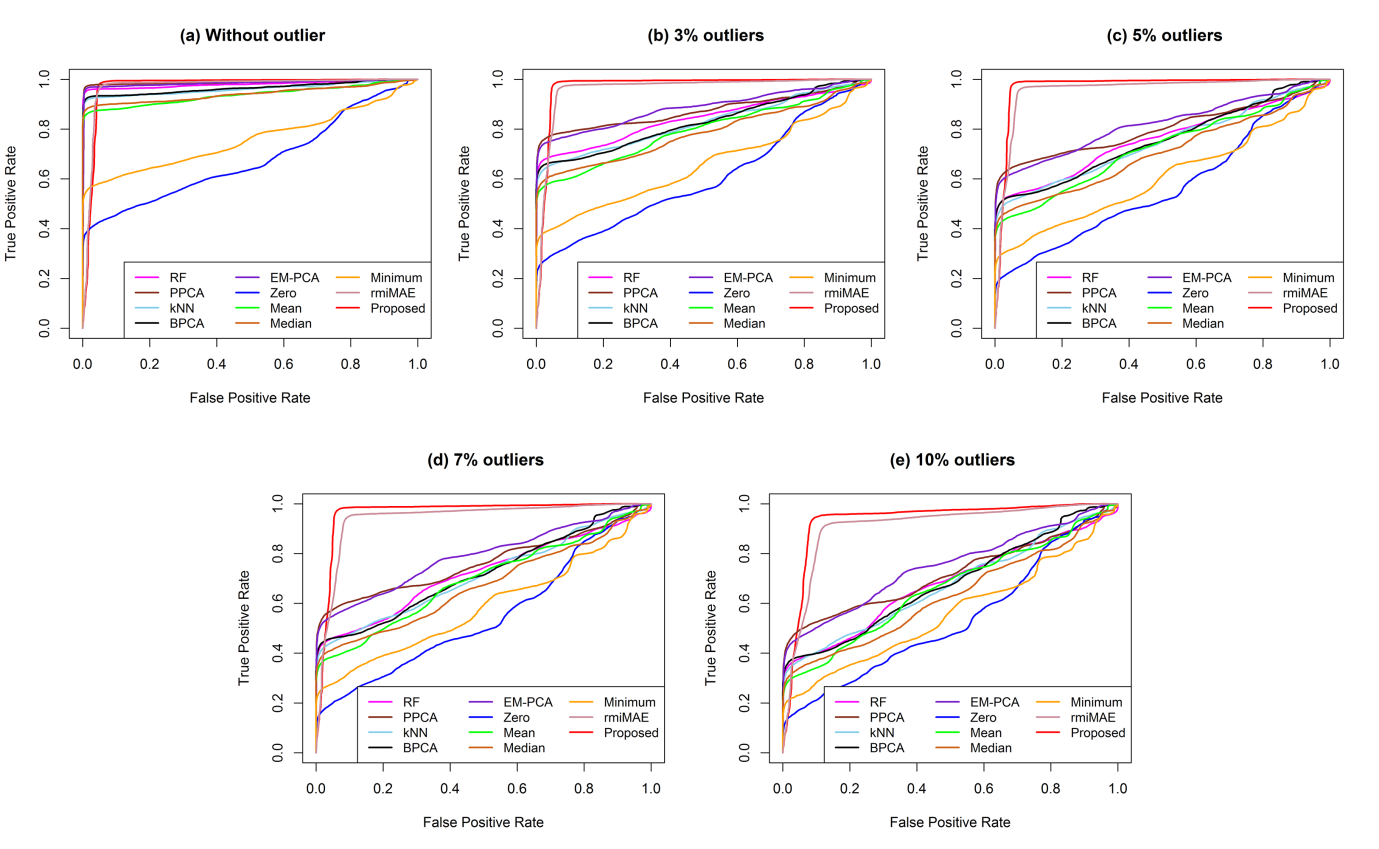


**Figure S10.** Performance investigation of different missing value imputation techniques using receiver operating characteristic curve of DE calculation for three class level dataset with 15% missing values in absence and presence of outliers.

**
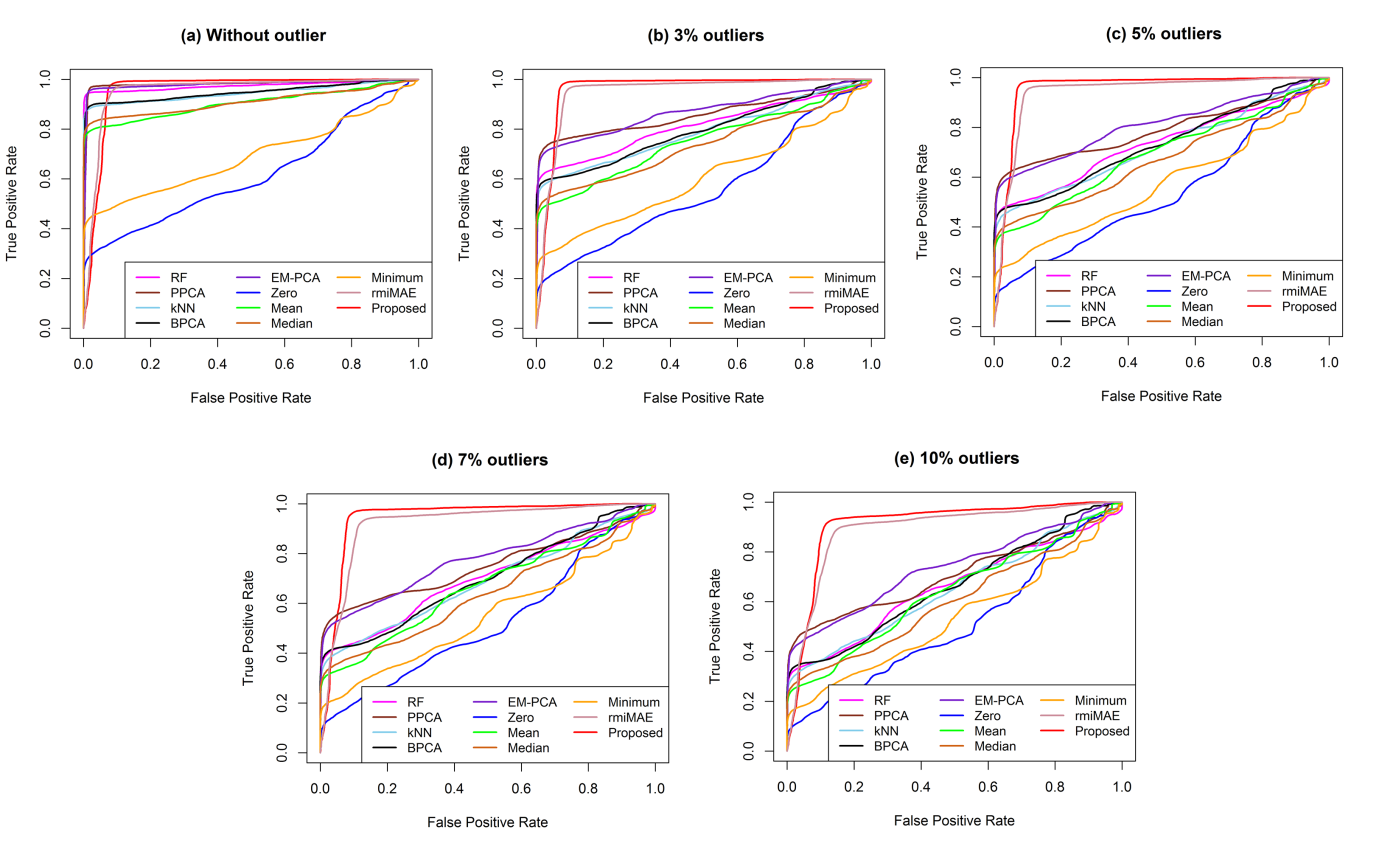
**

**Figure S11.** Performance investigation of different missing value imputation techniques using receiver operating characteristic curve of DE calculation for three class level dataset with 20% missing values in absence and presence of outliers.

**
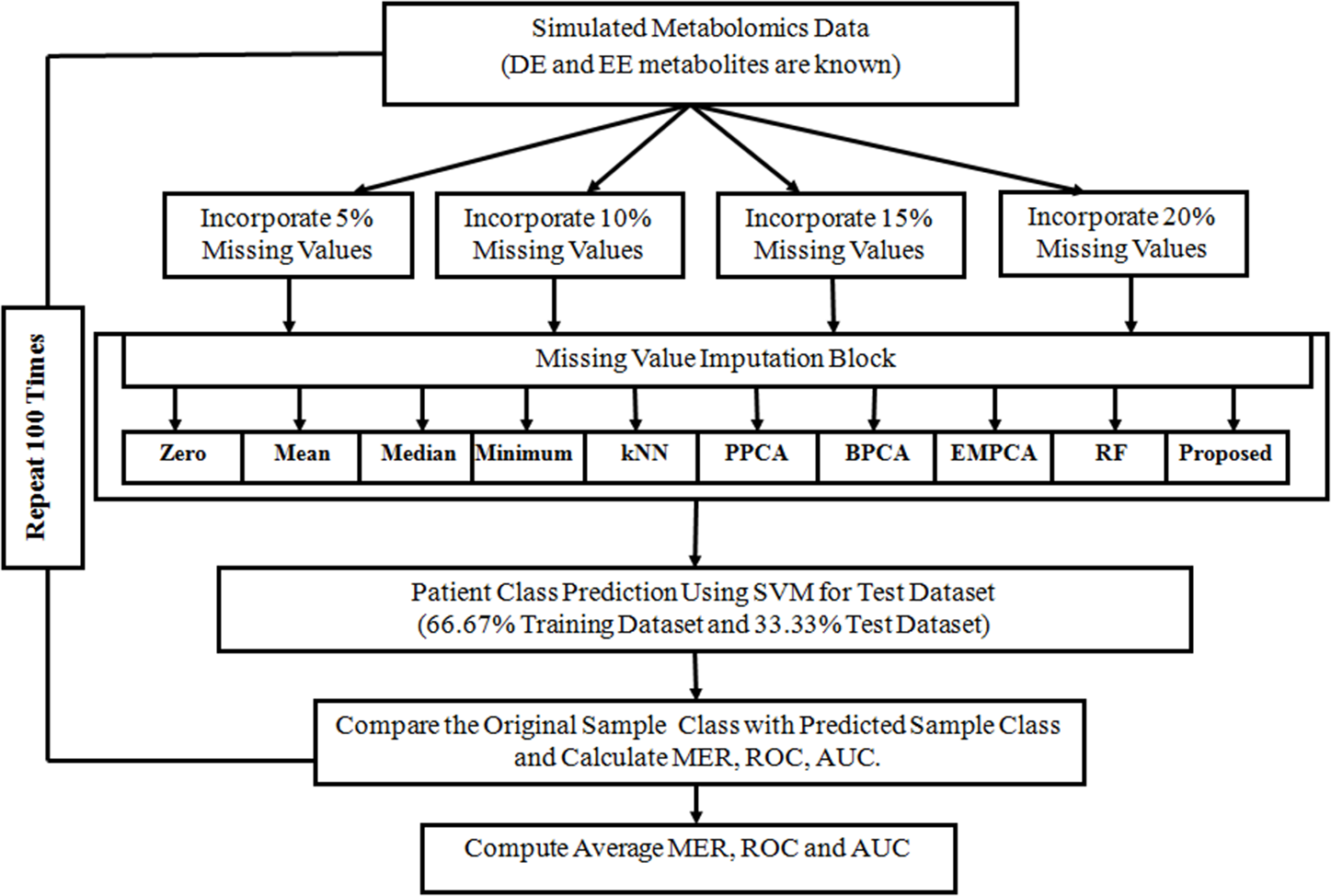
**

**Figure S12.**  Performance measures calculation procedure on the basis of sample classification.

**
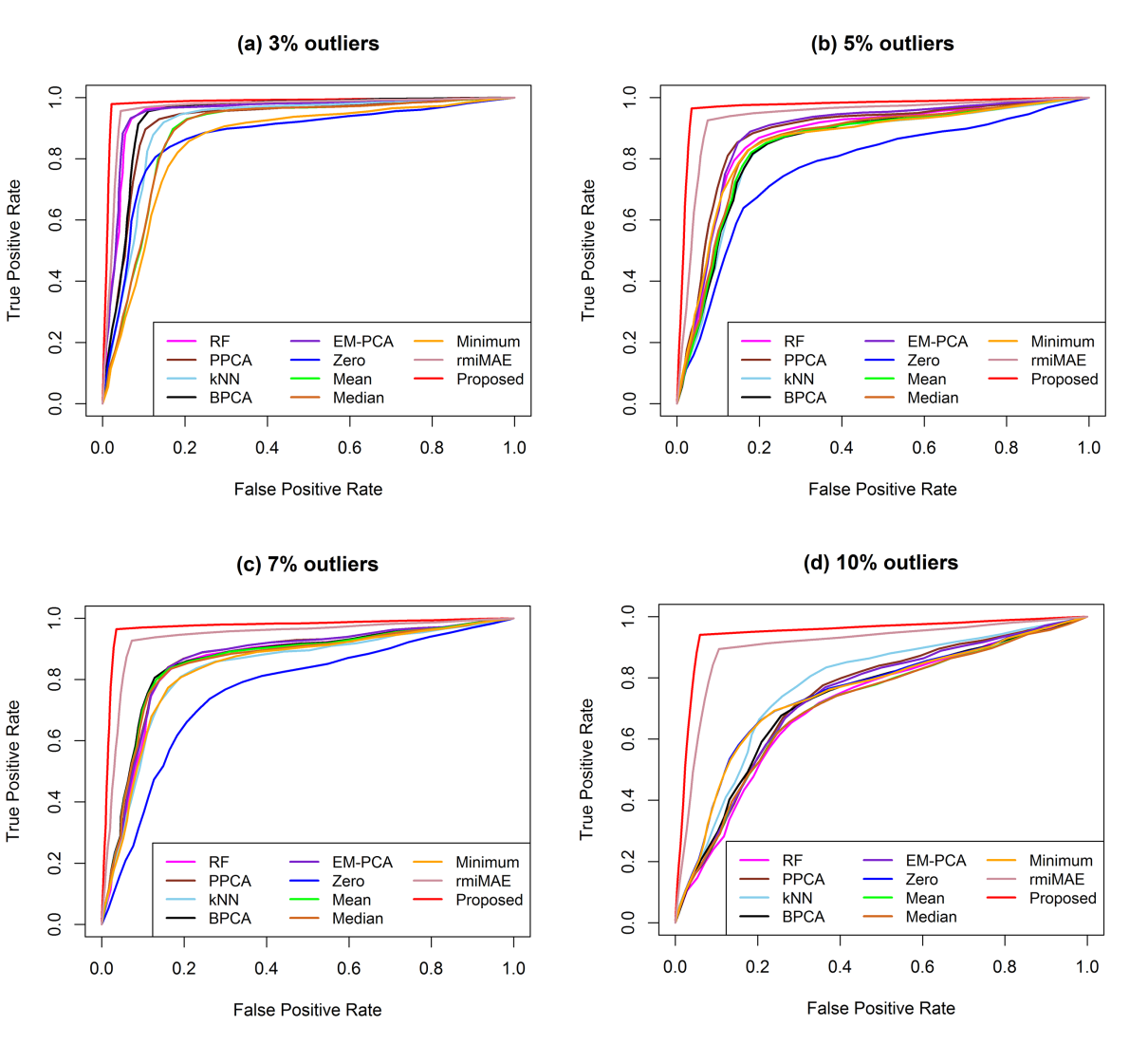
**

**Figure S13.** Performance investigation of different missing value imputation techniques using receiver operating characteristic curve of sample classification for two class level dataset with 10% missing values in presence of outliers.

**
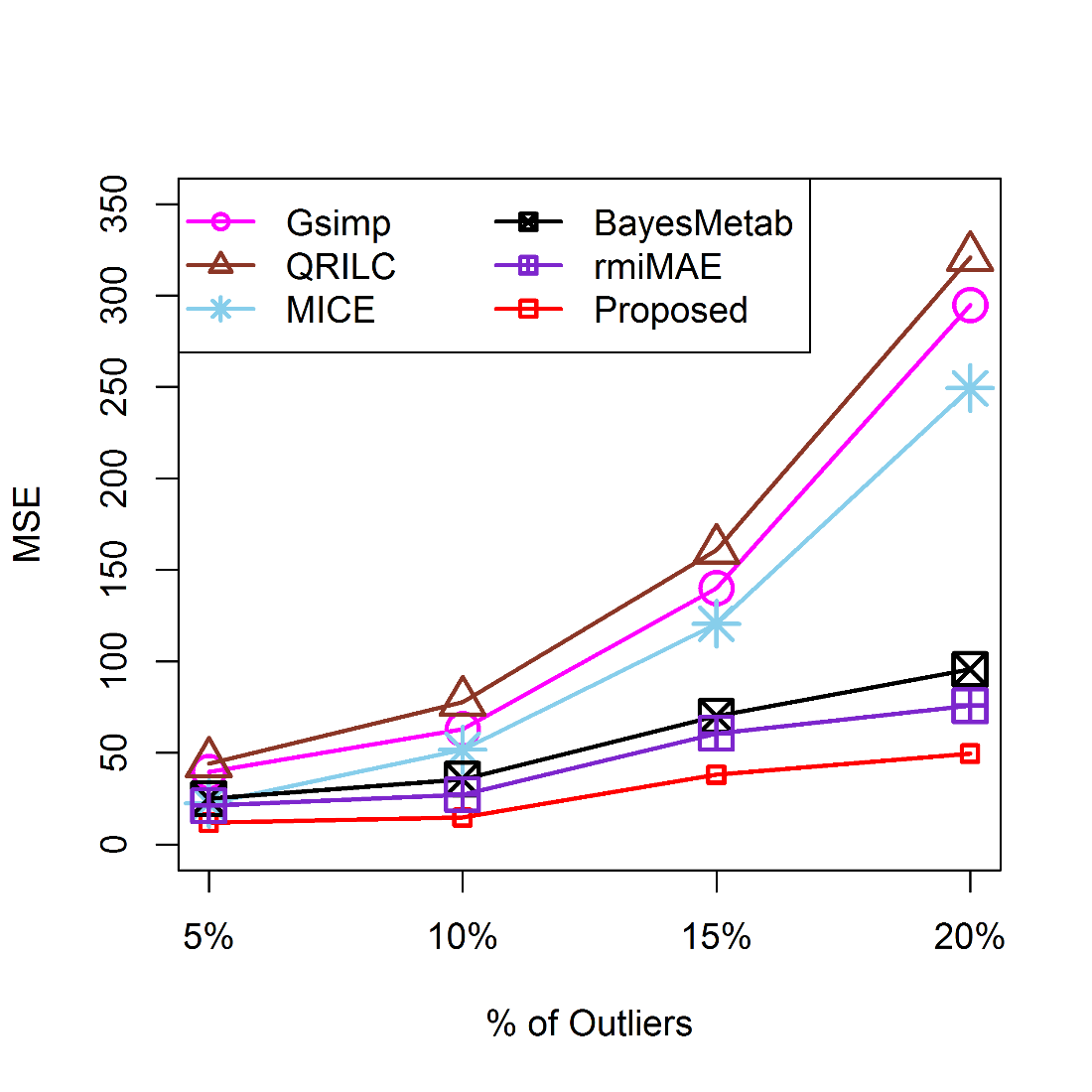
**

**Figure S14.** Performance investigation of different missing value imputation techniques using MSE calculation for different rates of missing values using human cachexia dataset
